# Oncogenic switch and single-agent MET inhibitor sensitivity in a subset of *EGFR*-mutant lung cancer

**DOI:** 10.1126/scitranslmed.abb3738

**Published:** 2021-09-01

**Authors:** Pınar Özden Eser, Raymond M. Paranal, Jieun Son, Elena Ivanova, Yanan Kuang, Heidi M. Haikala, Ciric To, Jeffrey J. Okoro, Kshiti H. Dholakia, Jihyun Choi, Yoonji Eum, Atsuko Ogino, Pavlos Missios, Dalia Ercan, Man Xu, Michael J. Poitras, Stephen Wang, Kenneth Ngo, Michael Dills, Masahiko Yanagita, Timothy Lopez, Mika Lin, Jeanelle Tsai, Nicolas Floch, Emily S. Chambers, Jennifer Heng, Rana Anjum, Alison D. Santucci, Kesi Michael, Alwin G. Schuller, Darren Cross, Paul D. Smith, Geoffrey R. Oxnard, David A. Barbie, Lynette M. Sholl, Magda Bahcall, Sangeetha Palakurthi, Prafulla C. Gokhale, Cloud P. Paweletz, George Q. Daley, Pasi A. Jänne

**Affiliations:** 1 Lowe Center for Thoracic Oncology, Dana-Farber Cancer Institute, Boston, MA 02215, USA.; 2 Department of Medical Oncology, Dana-Farber Cancer Institute, Boston, MA 02215, USA.; 3 Department of Medicine, Brigham and Women’s Hospital and Harvard Medical School, Boston, MA 02215, USA.; 4 Harvard Medical School, Boston, MA 02115, USA.; 5 Belfer Center for Applied Cancer Science, Dana-Farber Cancer Institute, Boston, MA 02215, USA.; 6 Stem Cell Program, Boston Children’s Hospital, Boston, MA 02115, USA.; 7 Experimental Therapeutics Core, Belfer Center for Applied Cancer Science, Dana-Farber Cancer Institute, Boston, MA 02215, USA.; 8 Oncology R&D, Bioscience, AstraZeneca, CRUK Cambridge Institute, Robinson Way, Cambridge CB2 0RE, UK.; 9 Bioscience, Oncology R&D, AstraZeneca, 25 Gatehouse Park, Waltham, MA 02451, USA.; 10 Global Medical Affairs, Oncology Business Unit, AstraZeneca, 136 Hills Road, Cambridge CB2 8PA, UK.; 11 Department of Pathology, Brigham and Women’s Hospital and Harvard Medical School, Boston, MA 02215, USA.; 12 Harvard Stem Cell Institute, Cambridge, MA 02138, USA.

## Abstract

The clinical efficacy of epidermal growth factor receptor (EGFR)–targeted therapy in *EGFR*-mutant non–small cell lung cancer is limited by the development of drug resistance. One mechanism of EGFR inhibitor resistance occurs through amplification of the human growth factor receptor (*MET*) proto-oncogene, which bypasses EGFR to reactivate downstream signaling. Tumors exhibiting concurrent *EGFR* mutation and *MET* amplification are historically thought to be codependent on the activation of both oncogenes. Hence, patients whose tumors harbor both alterations are commonly treated with a combination of EGFR and MET tyrosine kinase inhibitors (TKIs). Here, we identify and characterize six patient-derived models of *EGFR*-mutant, *MET*-amplified lung cancer that have switched oncogene dependence to rely exclusively on MET activation for survival. We demonstrate in this MET-driven subset of EGFR TKI-refractory cancers that canonical EGFR downstream signaling was governed by MET, even in the presence of sustained mutant EGFR expression and activation. In these models, combined EGFR and MET inhibition did not result in greater efficacy in vitro or in vivo compared to single-agent MET inhibition. We further identified a reduced *EGFR:MET* mRNA expression stoichiometry as associated with *MET* oncogene dependence and single-agent MET TKI sensitivity. Tumors from 10 of 11 EGFR inhibitor–resistant *EGFR*-mutant, *MET*-amplified patients also exhibited a reduced *EGFR:MET* mRNA ratio. Our findings reveal that a subset of *EGFR*-mutant, *MET*-amplified lung cancers develop dependence on MET activation alone, suggesting that such patients could be treated with a single-agent MET TKI rather than the current standard-of-care EGFR and MET inhibitor combination regimens.

## INTRODUCTION

The epidermal growth factor receptor (EGFR) is a cell surface receptor tyrosine kinase that promotes cell proliferation and survival through activation of downstream signaling cascades (1, 2). A subset of non–small cell lung cancer (NSCLC) is driven by activating somatic *EGFR* mutations, most commonly short in-frame deletions of exon 19 (del19) or missense mutations resulting in the amino acid substitution L858R. These activating mutations promote constitutive, ligand-independent receptor activation (3, 4) and typically confer de novo sensitivity to targeted EGFR tyrosine kinase inhibitors (TKIs) (5–7).

Although *EGFR*-mutant tumors initially respond to EGFR TKIs, the clinical success of these inhibitors is limited by the development of acquired drug resistance (8). Amplification of *MET*, the gene encoding the receptor tyrosine kinase hepatocyte growth factor receptor (HGFR, or MET) is a conserved mechanism of resistance to EGFR inhibitors, observed in 5 to 20% of patients who develop clinical resistance to treatment with EGFR TKIs (9–11). There is substantial cross-talk between signaling pathways downstream of EGFR and MET, including cascades culminating in the activation of the proto-oncogenic extracellular signal–regulated kinase (ERK1/2) and protein kinase B (Akt) (1, 2). This redundancy between signaling cascades gives rise to compensatory mechanisms that allow MET to bypass EGFR and reactivate downstream effectors, mediating resistance to EGFR-targeted therapies (12, 13).

The existing dogma dictates that *EGFR*-mutant and *MET*-amplified lung cancers codepend on the concurrent activation of both EGFR and a MET kinases for survival, making them unresponsive to single-agent TKI treatment against either receptor. Consequently, effective inhibition requires treatment with a combination of an EGFR and a MET TKI (8, 11). Small-molecule MET kinase inhibitors in clinical use or under clinical development include the kinase inhibitors crizotinib, savolitinib, capmatinib, and tepotinib (14–16). Multiple case reports and clinical trials have successfully combined EGFR and MET inhibitors as a clinical strategy to overcome *MET* amplification–mediated resistance to EGFR TKI (14, 15, 17, 18).

A model of MET-mediated resistance to EGFR TKIs is the HCC827GR6 cell line, which was established through long-term dose-escalation treatment of the parental EGFR-dependent HCC827 cell line with gefitinib (9). The EGFR/MET-codependent HCC827GR6 cells exhibit resistance to treatment with single-agent EGFR and MET inhibitors but retain sensitivity to combination EGFR and MET TKI treatment. Characterization of this in vitro model was the first evidence of *MET* amplification conferring resistance to EGFR TKIs and provided the preclinical basis for clinical trials evaluating the efficacy of combined EGFR and MET inhibition. Mechanistically, *MET* amplification in HCC827GR6 cells imparts resistance to EGFR TKI by bypassing EGFR through transphosphorylation and activation of the kinase-deficient EGFR family member human epidermal growth factor receptor 3 (HER3, or ERBB3) (9).

In our ongoing effort to develop *EGFR*-mutant, EGFR inhibitor–resistant patient-derived models, we identified several models harboring *MET* amplification that exhibited an unexpected sensitivity to single-agent MET inhibition. Here, we provide a mechanistic basis for these observations and propose a potential strategy to identify patients with *EGFR*-mutant cancer who may respond to single-agent MET TKI therapy.

## RESULTS

### Three patient-derived xenograft models of *EGFR*-mutant, *MET*-amplified NSCLC exhibit sensitivity to single-agent MET inhibitor treatment

**Fig. 1.**
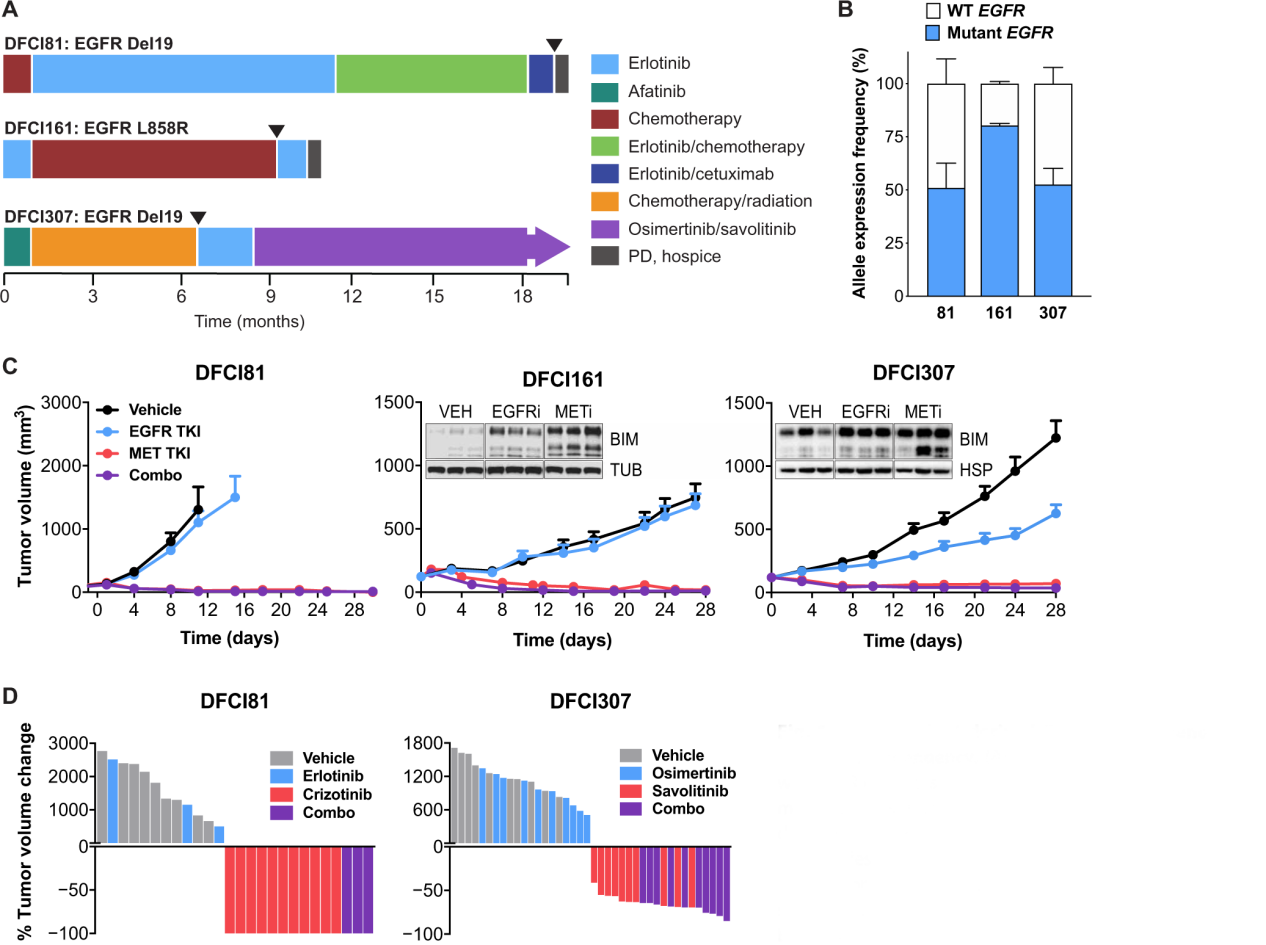
Three patient-derived *EGFR*-mutant xenograft models show MET dependency. (**A**) Treatment histories of patients treated with EGFR inhibitors. Arrows indicate time of specimen collection for model establishment. (**B**) Targeted NGS of cDNA showing patient-derived model expression of mutant compared to wild type (WT) *EGFR* alleles. Error bars indicate SDs of allele expression among three tumors from three independent xenografts for each model. (**C**) Response of DFCI81, DFCI161, and DFCI307 PDX models to single-agent EGFR inhibitors (EGFRi) or MET inhibitors (METi). Each data point represents means and SEM among *n* = 3 (DFCI81 and DFCI161, combination treatment), *n* = 8 to 12 (DFCI161, vehicle, erlotinib, and crizotinib), and *n* = 10 (DFCI307) mice per study arm. Inset: Western blots show up-regulation of BIM in DFCI161 and DFCI307 PDX tumors in response to TKI treatment. Loading controls shown for comparison are tubulin (TUB) and heat shock protein 90 (HSP). Tumors shown in Western blot studies were derived from three different mice per study arm. (**D**) Waterfall plots demonstrating maximal treatment response to single-agent MET or EGFR inhibitor or a combination of the two. Each bar is derived from the maximal response of a single tumor-bearing mouse to drug treatment.

We established three patient-derived xenograft (PDX) models of lung adenocarcinoma from the drug refractory tumors of patients who developed resistance to single-agent EGFR TKI therapy. One model, DFCI81, was established from the tumor-derived cell line of a patient who developed acquired resistance after an initial long-term response to treatment with EGFR-targeted therapy, whereas the other two models, DFCI161 and DFCI307, exhibited de novo resistance to EGFR TKI (Fig. 1A). Targeted next-generation sequencing (NGS; OncoPanel) (19) completed at the time of specimen collection revealed *EGFR*-activating mutations (exon 19 ELREA deletion in DFCI81; exon 19 LREAT deletion in DFCI307; exon 21 L858R point mutation in DFCI161), no evidence of *EGFR* secondary mutations, and genomic *MET* copy number gain in all three models (fig. S1A) (14). All three PDX models retained expression of the mutant *EGFR* allele, as determined by next-generation complementary DNA (cDNA) sequencing (Fig. 1B), and exhibited *MET* amplification by fluorescence in situ hybridization (FISH) analysis (fig. S1B) (20, 21).

Treatment of all three PDX models showed sensitivity to single-agent MET inhibition and resistance to single-agent EGFR inhibition in vivo. MET inhibition led to sustained tumor regression, and Western blot analysis revealed that MET inhibition alone induced up-regulation of the proapoptotic Bcl2-like protein 11 (BIM) in the tumor xenografts (Fig. 1C). We also treated all three models with the combination of an EGFR inhibitor (erlotinib for DFCI81 and DFCI161 and osimertinib for DFCI307) and a MET inhibitor (crizotinib for DFCI81 and DFCI161 and savolitinib for DFCI307). There was no evidence that the addition of an EGFR inhibitor improved MET inhibitor efficacy in any of the PDX models (Fig. 1, C and D). In both DFCI81 and DFCI307, the average tumor volume decrease was statistically indistinguishable between mice treated with a MET inhibitor alone and in combination with an EGFR inhibitor (fig. S1C). We also observed no difference in tumor outgrowth after drug cessation in mice treated with single-agent MET inhibitor versus with a combination of MET and EGFR inhibitors (fig. S1D). DFCI81 xenograft-bearing mice showed no tumor growth after 100 days of drug cessation after treatment with a single-agent MET inhibitor, whereas in the DFCI161 and DFCI307 models, tumor outgrowth after drug withdrawal was similar in mice treated with a MET inhibitor alone or in combination with an EGFR inhibitor (fig. S1D).

### *EGFR*-mutant patient-derived cell lines exhibit single-agent MET inhibitor sensitivity

**Fig. 2.**
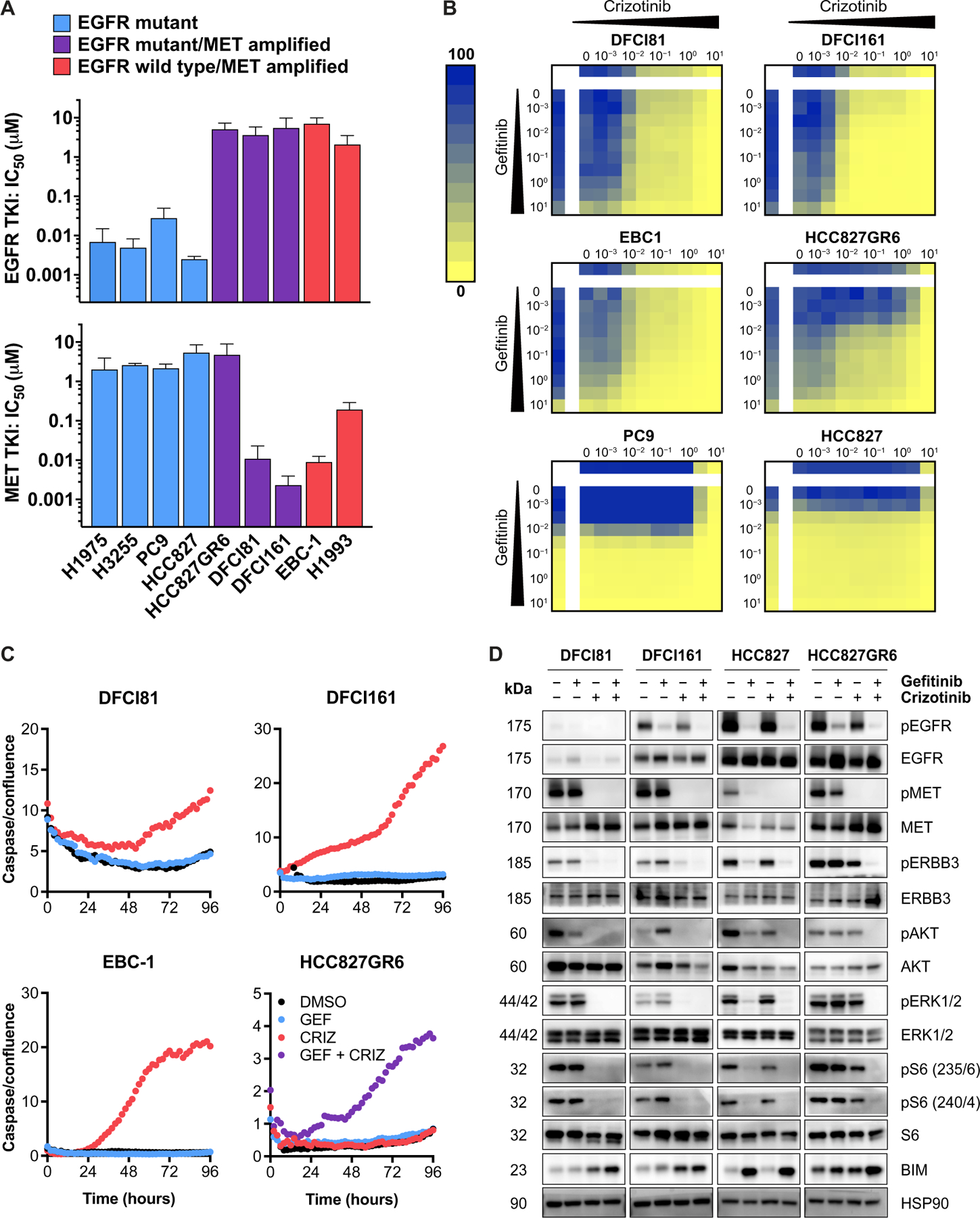
Patient-derived *EGFR*-mutant cell line models show MET dependency for survival and activation of canonical EGFR downstream signaling. (**A**) Efficacy of MET and EGFR inhibitors in EGFR-dependent (H1975, H3255, PC9, and HCC827), EGFR/MET-codependent (HCC827GR6), and MET-dependent (EBC-1, H1993, DFCI81, and DFCI161) cell lines. Each IC_50_ value was extrapolated from a nine-point dose curve (*n* = 6 replicates per dose) of TKI, with cell viability readouts after a 96-hour incubation in drug. Each bar represents mean IC_50_ values calculated from three independent biological replicate studies; error bars denote SD. (**B**) Drug concentration matrices showing percent viability after treatment with inhibitor dose gradients. Drug sensitivities of MET-dependent EBC-1 cells and EGFR-dependent parental HCC827 and PC9 cells are shown for comparison. Doses indicated are in micromolars. (**C**) Quantification of caspase 3/7 activation over a 96-hour time course of 1 μM TKI treatment. Data displayed are representative of three biological replicate studies. Fluorescence-quantified caspase 3/7 values are normalized to cell confluence for each time point. (**D**) Protein phosphorylation evaluated in MET-dependent DFCI81 and DFCI161 cells compared to control HCC827 and HCC827GR6 cells treated with vehicle control dimethyl sulfoxide (DMSO), 1 μM gefitinib, 1 μM crizotinib, or equimolar 1 μM combination of gefitinib and crizotinib to compare downstream activation of ERBB3, Akt, and ERK1/2 and expression of proapoptotic BIM after TKI treatment. Cells were lysed 16 hours after treatment. Western blot shows representative data from one of three replicate studies.

In addition to PDX models, we also established cell line models of DFCI81 and DFCI161. The drug sensitivity profiles of DFCI81 and DFCI161 cell lines were compared to a panel of EGFR-dependent, EGFR/MET-codependent, and MET-dependent models (Fig. 2A and fig. S2A). These studies revealed that the DFCI81 and DFCI161 cell lines were sensitive to MET inhibition, exhibiting crizotinib half-maximal inhibitory concentration (IC_50_) values in the nanomolar range (Fig. 2A). A panel of EGFR and MET inhibitors tested against DFCI81 and DFCI161 cell lines corroborated that both models are refractory to EGFR inhibitors and highly sensitive to all tested single-agent MET inhibitors (fig. S2A). We further treated a panel of cell lines with a drug concentration matrix to evaluate the added benefit, if any, of concomitant EGFR inhibition over MET inhibition alone (Fig. 2B). Our analysis revealed a lack of additive or synergistic effect of combining gefitinib with crizotinib in the DFCI81 and DFCI161 cell lines and acute sensitivity to single-agent MET inhibition to an extent comparable to the *EGFR* wild-type EBC-1 line. This sensitivity contrasted with the EGFR/MET codependence observed in the HCC827GR6 cells and the single-agent gefitinib sensitivity of the EGFR-dependent controls PC9 and HCC827 (Fig. 2B). To ensure that the noted fluctuations in our assay readout were reflective of cell viability, we repeated concentration matrix drug treatment followed by crystal violet staining of surviving cells and confirmed analogous single-agent crizotinib sensitivity in the DFCI81 and DFCI161 models (fig. S2B). We also corroborated the results of our concentration matrix study using osimertinib (EGFR TKI) and savolitinib (MET TKI) dose gradients and demonstrated that the sensitivity of DFCI81 and DFCI161 to single-agent MET TKI was independent of the MET inhibitor selected (fig. S2, C and D). The activity of the pan-ERBB inhibitor afatinib was comparable to that of gefitinib, showing minimal single-agent or combination efficacy in the MET-dependent DFCI81, DFCI161, and EBC-1 models and synergizing with the crizotinib in the EGFR/MET-codependent HCC827GR6 cell line. This absence of synergy was corroborated by low Bliss synergy scores in the MET-dependent models compared to the EGFR/MET-codependent cells (22, 23). By contrast, the ERBB2-specific inhibitor tucatinib induced neither single-agent efficacy nor target-specific synergy in combination with MET inhibitor in any of the models tested (fig. S3, A and B). Last, we observed that single-agent treatment with crizotinib was sufficient to induce apoptosis, as quantified over time by a fluorescent caspase 3/7 activation assay, in both DFCI81 and DFCI161 cell lines, consistent with our in vitro and in vivo findings (Fig. 2C). Up-regulation of caspase induction in DFCI161 and DFCI81 cells over time was inversely correlated with optically determined cell confluence and was normalized to confluence to account for reduction in cell numbers under drug-sensitive conditions (fig. S3C). After a 96-hour TKI treatment, the incidence of confluence-normalized cleaved caspase 3/7 was significantly (*P* < 0.05) increased by single-agent crizotinib treatment in DFCI81 and DFCI161 cells, compared to EGFR-dependent and EGFR/MET-codependent controls (fig. S3D). Moreover, we noted that single-agent crizotinib treatment was sufficient to achieve maximal caspase 3/7 activation, which was not substantially enhanced by the addition of gefitinib in the MET-dependent models (fig. S3E).

We next interrogated the effects of MET inhibition on downstream signaling pathways. In the gefitinib-sensitive *EGFR*-mutant HCC827 cells, activating phosphorylation of the downstream kinases ERK1/2 and Akt relied on EGFR activation and was inhibited by single-agent gefitinib treatment (Fig. 2D and fig. S4A). In the EGFR and MET–codependent cell line HCC827GR6, downstream kinases were activated by both EGFR and MET, and simultaneous inhibition of both receptors was required to inhibit ERBB3 phosphorylation and downstream signaling, consistent with our prior studies (9). By contrast, in the case of the *EGFR*-mutant MET-dependent cell lines DFCI81 and DFCI161, gefitinib had no effect on signaling, whereas treatment with crizotinib alone was sufficient to ablate both Akt and ERK1/2 phosphorylation and induce proapoptotic BIM up-regulation. We also observed MET-dependent regulation of two phosphorylation sites of ribosomal protein S6, a downstream effector regulated by ERK1/2 and Akt activity, in both DFCI81 and DFCI161 cell lines (Fig. 2D) (24). DFCI307 PDX tumors treated in vivo with the EGFR TKI alone, MET TKI alone, or both drugs in combination similarly revealed that MET inhibition alone was sufficient to inhibit downstream Akt and ERK1/2 phosphorylation (fig. S4, B and C).

### MET-mediated ERBB3 activation predicts MET dependency in *EGFR*-mutant, *MET*-amplified models

**Fig. 3.**
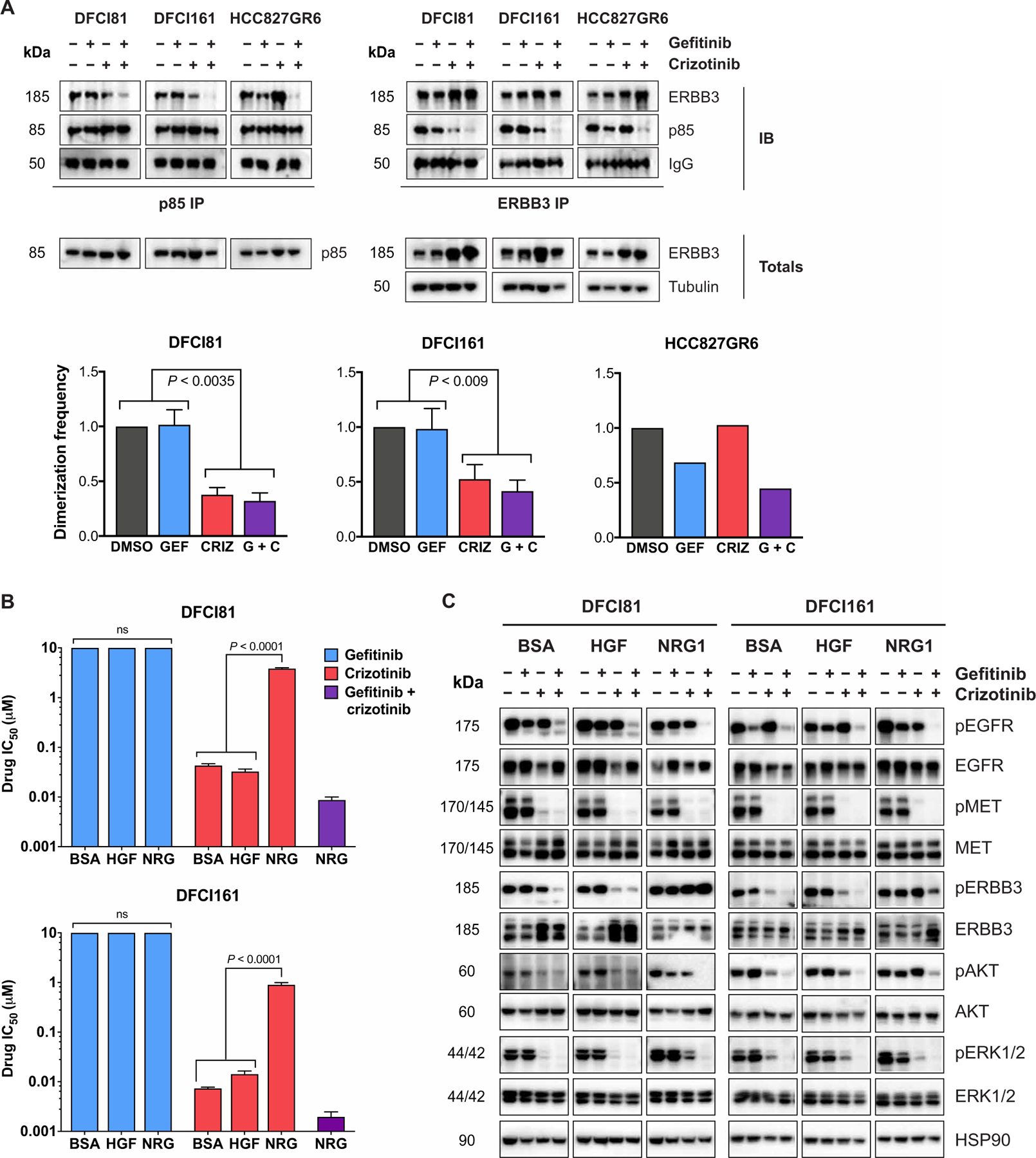
MET kinase is the predominant activator of ERBB3 signaling in *EGFR*-mutant, MET-dependent NSCLC. (**A**) Coimmunoprecipitation to assess the interaction between ERBB3 and the regulatory p85 subunit of PI3K after treatment with single-agent and combined EGFR and MET inhibitors. Complex formation was compared in MET-dependent DFCI81 and DFCI161 cells versus EGFR/MET-codependent HCC827GR6 cells. Dimerization frequencies were calculated by assessing the average intensity of ERBB3 immunoprecipitation (IP)–p85 immunoblot (IB) and normalizing to the average intensity of ERBB3 IP–ERBB3 IB for each pulldown. IgG, immunoglobulin G. (**B**) Sensitivity to gefitinib, crizotinib, and combination treatment was quantified in DFCI81 and DFCI161 cells treated in the presence of vehicle bovine serum albumin (BSA), recombinant MET ligand HGF, or recombinant ERBB3 ligand NRG1. Cells were seeded in recombinant ligand (10 ng/ml) and treated the next day. IC_50_ values were extrapolated from a nine-point dose curve (*n* = 6 replicates per dose) after 96 hours of TKI treatment. (**C**) Activation of downstream signaling pathways was compared after single-agent and combination EGFR and MET TKI treatment in the presence of BSA, HGF, or NRG1. Cells were plated in recombinant ligand (50 ng/ml), treated the following day with 1 μM individual or combined TKIs, and lysed and analyzed after 16 hours of incubation in inhibitor. Bar graphs show pooled data from three biological replicate studies in DFCI81 and DFCI161 cells. Statistical significance was determined by ANOVA, followed by Tukey’s posttest for multiple comparisons. ns, not significant.

In addition to the activation of downstream kinases ERK1/2 and Akt, we noted that phosphorylation of ERBB3 was inhibited by single-agent crizotinib treatment alone in DFCI81 and DFCI161 cell line models, in contrast to HCC827 and HCC827GR6 cells (Fig. 2D). Phosphorylation of ERBB3 has previously been shown to be regulated by EGFR in EGFR-dependent models (including HCC827) and to be codependent on EGFR and MET kinases for activation in EGFR/MET-codependent cells (HCC827GR6) (9). Because ERBB3 is an essential intermediary protein in the signal transduction from receptor tyrosine kinases to their downstream effectors, its phosphorylation is strongly predictive of oncogene dependency (Fig. 2D and fig. S4A). We postulated that ERBB3 may also be the principal effector of single-agent MET dependency in DFCI81 and DFCI161 and thereby that MET inhibition would disrupt the participation of ERBB3 in activated downstream signaling complexes. We tested this hypothesis by examining the effects of TKI treatment on the physical interaction between ERBB3 and its primary downstream effector, phosphoinositide 3-kinase (PI3K). Total ERBB3 expression in DFCI81 and DFCI161 cells increased after MET inhibition, a compensatory mechanism reported in other receptor tyrosine kinase–driven cancers treated with TKI (25, 26). Consequently, relative prevalence of ERBB3-p85 complexes was determined through normalization to quantity of immunoprecipitated ERBB3, which varied with treatment. In HCC827GR6 cells, ERBB3 protein coimmunoprecipitated with the p85 regulatory subunit of PI3K, an association that was disrupted only after combination treatment with both an EGFR and a MET kinase inhibitor, consistent with prior observations (9). In contrast, single-agent MET inhibitor treatment in the DFCI81 and DFCI161 cells was sufficient (*P* < 0.0035 and *P* < 0.009, respectively) to diminish the prevalence of ERBB3-p85 complexes (Fig. 3A). Collectively, these findings suggest that in the MET-dependent *EGFR*-mutant models, MET activates downstream signaling through ERBB3 phosphorylation. MET inhibition is also associated with disruption of both MET-ERBB3 and EGFR-ERBB3 dimers in DFCI81 and DFCI161 models, whereas EGFR TKI diminishes EGFR-ERBB3 dimerization in the EGFR-dependent HCC827 parental cells and EGFR/MET-codependent HCC827GR6 cells (fig. S5, A and B). Compensatory up-regulation of ERBB3 expression upon MET inhibition diminishes the magnitude of observed down-regulation of the ERBB-MET interaction in DFCI81 cells. Although the quantity of MET that coimmunoprecipitates with ERBB3 is not visibly down-regulated in DFCI161 cells, increased ERBB3 expression and immunoprecipitation are once more observed, indicating that a reduced proportion of the immunoprecipitated ERBB3 associates with MET under the crizotinib-treated condition relative to vehicle control. Accounting for ERBB3 up-regulation, single-agent gefitinib treatment does not substantially affect the prevalence of MET-ERBB3 dimers nor does it abolish dimerization observed between ERBB3 and EGFR. The ERBB3-EGFR interaction in DFCI81 cells was more sensitive to MET inhibition compared to EGFR inhibitor treatment. By contrast, EGFR-ERBB3 heterodimerization declines substantially after gefitinib treatment in EGFR-dependent HCC827 and EGFR/MET-codependent HCC827GR6 cells. Although these trends are compelling, they are not as pronounced as the reduction of ERBB3-p85 dimeric complexes because receptor tyrosine kinase dimerization interactions are not contingent upon protein phosphorylation, and the existence of inactive dimers may confound the results of coimmunoprecipitation readouts (27–29).

Although EGFR is expressed and phosphorylated in DFCI81 and DFCI161 cells, its expression and activation did not have any bearing on the phosphorylation of ERBB3 and appeared uncoupled from activation of downstream signaling in this context. This observation is unusual because EGFR is known to freely dimerize with ERBB3 across different models, both physiological and pathological (9, 30). To investigate this dichotomy, we treated DFCI81 and DFCI161 cells with exogenous ERBB3 ligand neuregulin (NRG1) to induce EGFR-ERBB3 heterodimerization in vitro (30) and assessed whether this stimulation induced MET-independent ERBB3 activation and, in turn, EGFR/MET codependency. NRG1 cotreatment induced crizotinib resistance in both DFCI81 and DFCI161 cell lines; however, both cell lines retained sensitivity to combination treatment with crizotinib and gefitinib (Fig. 3B), corroborating that NRG1 artificially recapitulates EGFR/MET codependence. Addition of the MET ligand hepatocyte growth factor (HGF) had no impact on single-agent crizotinib sensitivity. Examination of downstream signaling in MET TKI–treated DFCI81 and DFCI161 cells revealed that NRG1 promoted sustained phosphorylation of ERBB3 and downstream kinases ERK1/2 and Akt. Moreover, this downstream signaling was activated by EGFR in the presence of NRG1, because combination treatment with EGFR and MET TKI was necessary to ablate downstream signaling (Fig. 3C and fig. S6, A and B). These observations introduce the possibility that high concentrations of NRG1 but not HGF in the tumor microenvironment alter the drug sensitivity profiles in a subset of patients. Paracrine growth factor–mediated resistance to TKI has previously been studied (31, 32) and, in some cases, may be attributed to tumor-intrinsic NRG1 overexpression invoked by oncogenic NRG1 genomic fusions (33, 34). None of the MET-dependent patient-derived models we studied harbored activating NRG1 fusions; we thus focused on investigating cell-intrinsic mechanisms underlying MET dependence in *EGFR*-mutant models.

Observing that ligand-stimulated ERBB3 activation elicited EGFR-MET codependence in DFCI81 and DFCI161 cells, we next sought to assess whether ectopic overexpression of ERBB3 phenocopied this drug sensitivity shift. Because ERBB3 has low kinase activity, we anticipated that its overexpression would be insufficient to alter drug sensitivity. DFCI81 cells, which exhibit lower EGFR expression, were selected to test this hypothesis. DFCI81 cells transduced to constitutively overexpress ERBB3 did not shift away from gefitinib resistance and crizotinib sensitivity (fig. S6C), supporting the hypothesis that ERBB3 dimerizing and activating partner preference is not determined by ERBB3 expression.

### *EGFR*-mutant, MET-dependent models exhibit a reduced *EGFR:MET* mRNA expression ratio

**Fig. 4.**
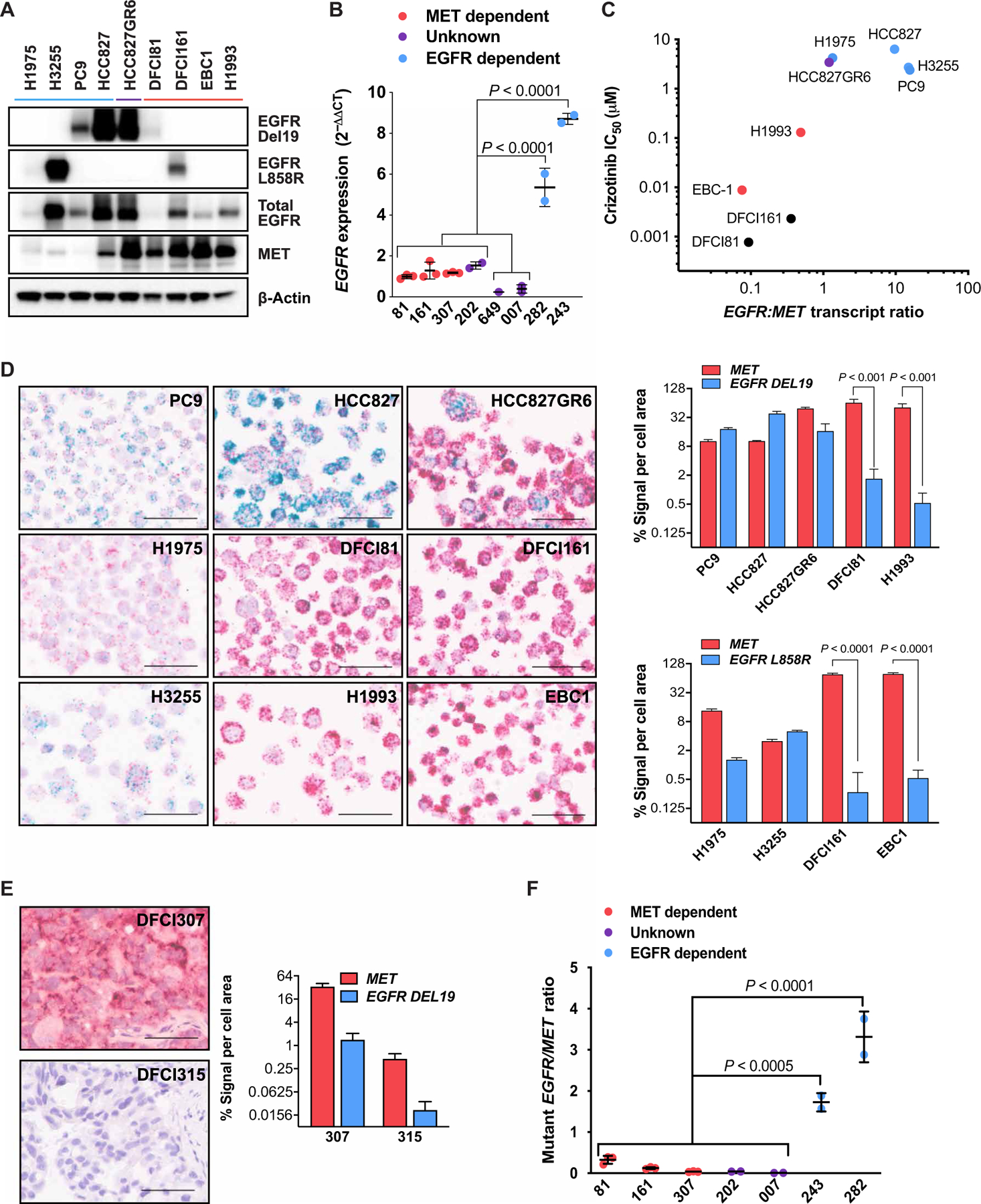
Association of *EGFR:MET* expression ratio with oncogene dependence. (**A**) Wild-type and mutant EGFR protein expression levels compared across MET-dependent cell lines including DFCI81 and DFCI161 (lanes indicated in red), EGFR-dependent control cell lines (blue), and EGFR-MET–codependent HCC827GR6 cells (purple). (**B**) *EGFR* mRNA in MET-dependent DFCI81, DFCI161, and DFCI307 PDX models compared to EGFR-dependent DFCI282 and DFCI243 xenograft models. *EGFR* mRNA expression was determined by qPCR in three additional *EGFR*-mutant primary NSCLC models (MR007, DFCI649, and DFCI202). Each data point represents the mean of three technical replicates from an untreated or vehicle-treated biological replicate (separate tumor or cell pellet). Statistical significance was determined by one-way ANOVA and Tukey’s multiple comparisons posttest. (**C**) Association of single-agent MET inhibitor IC_50_ with ratio of total *EGFR:MET* mRNA transcript expression. Each data point shows the mean of three technical replicates, with data representative of three replicate studies. (**D**) BaseScope in situ mRNA hybridization images of cell pellets, with red probe complementary to total *MET* mRNA and blue probe specific to mutant *EGFR* (ELREA exon 19 deletion for PC9, HCC827, HCC827GR6, DFCI81, and H1993; L858R for H1975, DFCI161, H3255, and EBC-1). For each BaseScope image, scale bars indicate a length of 50 μm, and quantification plots show means and SD of cell signal area of four representative fields per image. Significance was determined by paired *t* test to compare mutant *EGFR* to *MET* transcript expression for each cell line. (**E**) BaseScope imaging and quantification of *MET* and mutant *EGFR* (LREAT deletion) expression in the DFCI307 PDX model compared to a control *EGFR* wild-type, ERBB2-driven PDX model DFCI315. Scale bars indicate a length of 50 μm, and graphs show means and SD of quantified cell signal area of four representative fields per image. (**F**) Mutant *EGFR:MET* transcript ratios of models from (B) using RT-ddPCR analysis. Statistical significance was determined by one-way ANOVA and Tukey’s multiple comparisons posttest.

Having established and characterized models of single-agent MET dependency in *EGFR*-mutant NSCLC, we next sought to understand why, despite expression of the mutant *EGFR* allele, these models were sensitive to single-agent MET inhibitors. We noted that in both DFCI81 and DFCI161 cells, the degree of EGFR expression appeared lower compared to HCC827 or HCC827GR6 cells. To evaluate whether this observation was consistent across the genotypes of interest, we compared the expression of total and mutant EGFR and MET across a broader panel of lung cancer cell lines and xenografts, including EGFR-dependent models (H1975, H3255, PC9, and HCC827), EGFR/MET-codependent cells (HCC827GR6), and MET-dependent cells (EBC-1 and H1993) (Fig. 4A and fig. S6D). We noted that EGFR expression was generally, although not universally, higher in EGFR-dependent or codependent models compared to MET-dependent cell lines. Similarly, we observed greater total *EGFR* mRNA expression in *EGFR*-mutant, EGFR inhibitor–sensitive PDX models DFCI243 (*EGFR* del19/T790M) and DFCI282 (*EGFR* L858R/T790M) (35–37) than in the MET inhibitor–sensitive *EGFR*-mutant DFCI81, DFCI161, and DFCI307 xenografts (Fig. 4B).

Although the observation of decreased EGFR expression was generally predictive of MET dependency in our cell line and PDX models, this may be difficult to translate into a clinical assay to identify *EGFR*-mutant patients likely to benefit from single-agent MET inhibition. For instance, the EGFR-dependent H1975 cells (*EGFR* L858R/T790M) exhibited lower *EGFR* expression, confounding our observation (Fig. 4). To reflect the biology underlying the relationship between protein expression stoichiometry and oncogene dependency, we incorporated *MET* expression as a denominator, assessing the *EGFR:MET* transcript ratio across our panel of NSCLC models. A lower *EGFR:MET* ratio directly associated with MET inhibitor sensitivity, whereas a higher ratio was associated with single-agent EGFR inhibitor sensitivity or EGFR/MET codependence (Fig. 4C).

To develop an assay that could be used to evaluate clinical formalin-fixed paraffin-embedded (FFPE) tumor specimens, we evaluated *EGFR:MET* transcript ratios through BaseScope, an RNA-based in situ hybridization assay in which fluorescent signal area acts as a surrogate for RNA transcript expression. We designed mutant-specific RNA in situ hybridization probes to enable quantification of the relative expression of activating mutant *EGFR* in our models. We first generated nine cell lines to mimic clinical specimens and performed BaseScope analysis. Consistent with our reverse transcription quantitative polymerase chain reaction (RT-qPCR) analysis, the signal area of *MET* compared to mutant *EGFR* counts per cell was higher in DFCI81 and DFCI161 cells (*P* ≤ 0.001 and *P* < 0.0001, respectively; Fig. 4D). Although the EGFR/MET-codependent HCC827GR6 cells and EGFR-dependent H1975 cells exhibited lower *EGFR* signal area compared to the other EGFR-dependent models, they retained significantly (*P* < 0.025) higher *EGFR:MET* ratios by BaseScope analysis compared to both *EGFR*-mutant MET-dependent DFCI81 and DFCI161 cells and *EGFR* wild-type MET-dependent EBC-1 and H1993 cells (fig. S7A). We next compared the *EGFR:MET* transcript ratios of our confirmed MET-dependent DFCI307 PDX model to the *EGFR* wild-type control xenograft DFCI315 (*HER2* exon20 V777_G778insGSP) (Fig. 4E) (37) and observed that the DFCI307 cells exhibited a statistically indistinguishable mutant *EGFR:MET* signal ratio compared to the DFCI315 xenografts (fig. S7B).

To optimize a more clinically deployable assay, we cross-referenced the RT-qPCR–based and BaseScope-based *EGFR:MET* transcript ratios against results obtained by an RT droplet digital PCR (RT-ddPCR) assay. Using our existing ddPCR assay platform (38), we designed mRNA-complementary primers specific for *EGFR* L858R and *EGFR* del19 (both ELREA and LREAT), as well as wild-type *MET* (table S1). Applying this assay to fresh PDX tumors, we corroborated a markedly low mutant *EGFR:MET* transcript ratio in the MET-dependent DFCI81, DFCI161, and DFCI307 tumors compared to the EGFR-dependent control tumors DFCI243 and DFCI282 (Fig. 4F and fig. S7C). To model the stability of *EGFR* and *MET* mRNA in FFPE clinical specimens, we compared the ddPCR mutant *EGFR:MET* ratios from three fresh PDX tumors to those derived from mRNA of the same tumors after formalin fixation/paraffin embedding (fig. S7D).

### Identification of three additional patient-derived, *EGFR*-mutant, *MET*-dependent models exhibiting low *EGFR:MET* mRNA expression ratio

**Fig. 5.**
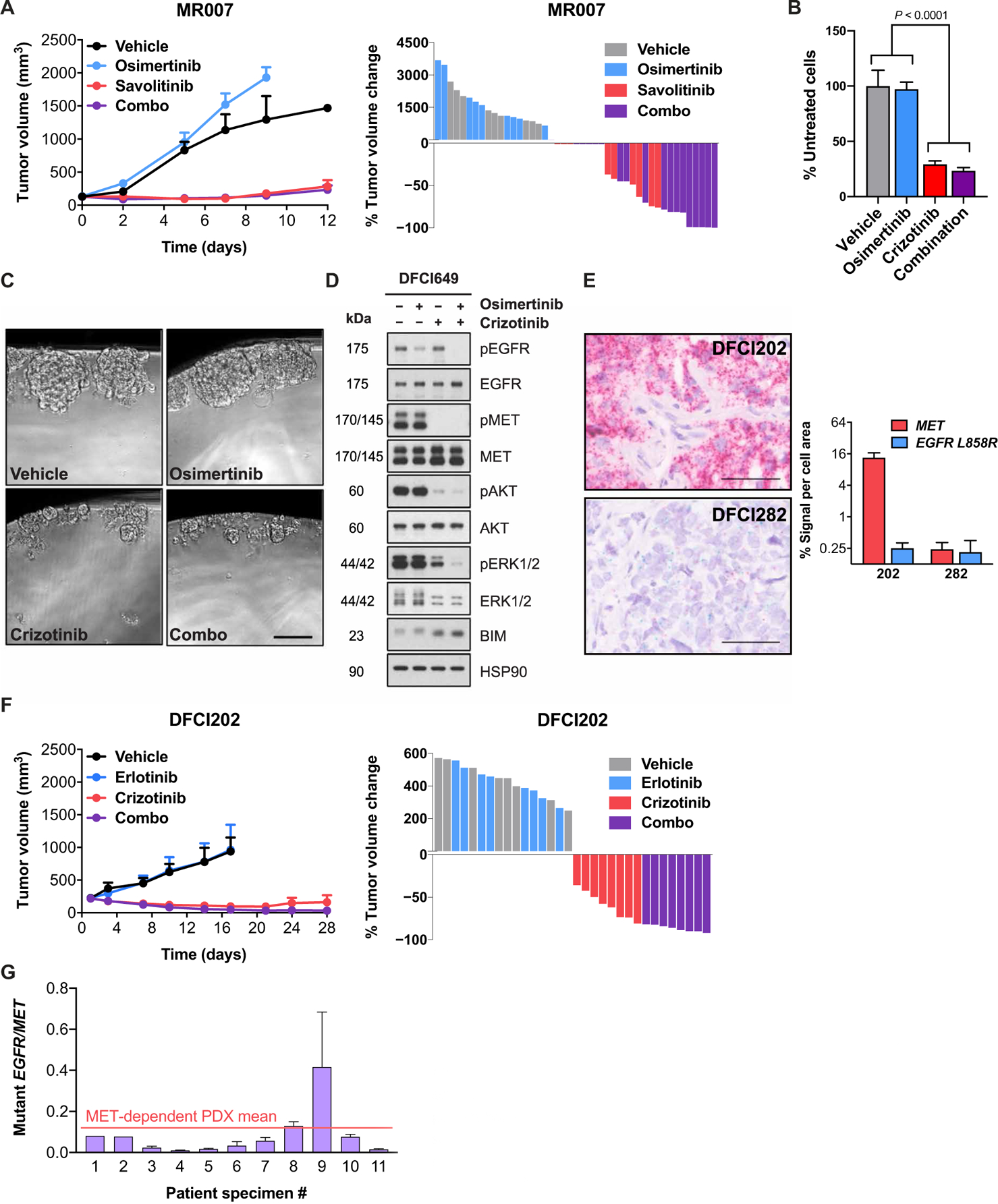
Characterization of oncogene dependence in three additional primary NSCLC models harboring concurrent *EGFR* mutation and *MET* amplification. (**A**) Comparative sensitivity of MR007, an in vivo xenograft model established from the tumor of a post-osimertinib patient harboring *EGFR* L858R and *MET* amplification (39), to single-agent EGFR TKI osimertinib, single-agent MET TKI savolitinib, or a combination of both inhibitors. Each data point represents means and SEM among *n* = 9 (vehicle, osimertinib, and savolitinib) or *n* = 18 (combination) mice per study arm. (**B**) Single-agent and combined TKI sensitivity of DFCI649, an organoid model established from the tumor of an *EGFR*-mutant, *MET*-amplified patient. Each IC_50_ value was extrapolated from a nine-point TKI dose curve (*n* = 6 replicates per dose), with cell viability readouts after a 96-hour incubation in drug. Each bar represents the mean of IC_50_ values calculated from independent biological replicate studies; error bars denote SD. Statistical significance was determined by one-way ANOVA, followed by Tukey’s posttest for multiple comparisons. (**C**) Images showing DFCI649 organoids after a 96-hour drug treatment. Photos were taken at equal magnification. Scale bar, 50 μm. (**D**) Activation state of canonical EGFR downstream signaling in DFCI649 cells in response to treatment with 1 μM osimertinib, 1 μM crizotinib, or a combination of both TKIs. (**E**) BaseScope imaging and quantification of *MET* and *EGFR* L858R transcript abundance in the patient-derived DFCI202 PDX model compared to control EGFR-driven PDX model DFCI282. For each BaseScope image, scale bars indicate a length of 50 μm, and quantification graphs show means and SD of cell signal area from four representative fields per image. (**F**) Single-agent crizotinib sensitivity of DFCI202, a PDX model established from a de novo erlotinib-resistant tumor harboring concurrent *EGFR* L858R mutation and *MET* amplification. (**G**) RT-ddPCR quantification of mutant *EGFR*–to–total *MET* expression ratio reveals a range of values across EGFR inhibitor–resistant, *EGFR*-mutant, *MET*-amplified patient specimens.

We next sought to test whether low *EGFR:MET* expression ratio was predictive of MET dependency in three additional patient-derived *EGFR*-mutant, *MET*-amplified NSCLC models: MR007, DFCI202, and DFCI649. The MR007 PDX model was developed from an osimertinib-resistant *EGFR*-mutant patient whose drug refractory tumor harbored *MET* amplification and was found to be sensitive to single-agent savolitinib in vivo (Fig. 5A) (39). Addition of osimertinib to savolitinib did not substantially improve the in vivo efficacy of single-agent savolitinib treatment (Fig. 5A and fig. S7E). Retrospective analysis of *EGFR* expression in MR007 by both RT-qPCR and RT-ddPCR revealed reduced *EGFR* transcript expression and a decreased *EGFR:MET* transcript ratio in the PDX model (Fig. 4, B and F, and fig. S7F).

DFCI649 is a patient-derived organoid model generated from the *EGFR*-mutant, *MET*-amplified tumor of an osimertinib-resistant patient (fig. S7, G and H). This model was sensitive to single-agent MET inhibition, with no significant benefit of concomitant EGFR inhibition (Fig. 5, B and C). The magnitude of single-agent crizotinib sensitivity and osimertinib resistance of the DFCI649 organoid model were comparable to a PDX-derived three-dimensional organoid model of the MET-dependent DFCI81 cell line (fig. S7I). Similar to DFCI81 and DFCI161 cell lines, MET inhibition alone in the DFCI649 organoid model was sufficient to diminish downstream Akt and ERK1/2 phosphorylation and induce BIM up-regulation to a degree comparable to that achievable by EGFR/MET TKI combination treatment (Fig. 5D and fig. S8, A and B). Exposure of the DFCI649 organoid model to drug concentration matrices corroborated single-agent sensitivity to MET inhibitors, with negligible additive effect of high-dose treatment with EGFR or ERBB family TKI (fig. S8, C and D). As with MR007, retrospective RT-qPCR analysis showed low *EGFR* transcript expression in DFCI649 cells, coinciding with the quantities we observed in other *EGFR*-mutant, MET-dependent NSCLC models (Fig. 4B).

DFCI202 is a PDX model derived from a de novo erlotinib-resistant patient whose *EGFR*-mutant tumor harbored concurrent *MET* amplification (fig. S9, A and B). Without prior insight into the drug sensitivity of the DFCI202 model, we observed that the PDX tumor exhibited a low total *EGFR* transcript expression as determined by qPCR (Fig. 4B) and a significantly (*P* < 0.0001) lower *EGFR* L858R:*MET* signal ratio compared to the EGFR-dependent DFCI282 PDX model evaluated by BaseScope (Fig. 5E and fig. S9C). We additionally observed a low mutant *EGFR:MET* expression ratio by RT-ddPCR (Fig. 4F and fig. S9D). The similarity of the *EGFR:MET* transcript ratio in DFCI202 to those of our other MET-dependent models suggested that DFCI202 could represent an additional *EGFR*-mutant, *MET*-amplified NSCLC model sensitive to single-agent MET inhibition. To test this hypothesis, we treated DFCI202 xenografts with single-agent crizotinib, single-agent erlotinib, or the combination of both agents (Fig. 5F). The DFCI202 PDX tumors showed an immediate and sustained sensitivity to single-agent crizotinib treatment but were unaffected by single-agent erlotinib treatment. There was no significantly enhanced efficacy in vivo with the addition of an EGFR TKI to the MET inhibitor (Fig. 5F and fig. S9E).

### Quantification of the *EGFR:MET* expression ratio in patient specimens

We next assessed mutant *EGFR* and total *MET* transcript expression in a series of *EGFR*-mutant patient tumor specimens (table S2) through quantitative RT-ddPCR. All specimens were from patients who had developed clinical resistance to EGFR inhibitor treatment and whose resistant cancers demonstrated *MET* amplification as defined by targeted NGS or FISH. One of these models (sample 2) was derived from the tumor of a treatment-naïve patient. Although we observed a range of mutant *EGFR:MET* expression ratios among the patient tumors, the majority (10 of 11) revealed low mutant *EGFR:MET* transcript ratios, within the same range as observed in our patient-derived models (Fig. 5E). One tumor, sample 9, exhibited a higher mutant *EGFR:MET* expression ratio compared to other patient specimens and relative to the mean mutant *EGFR:MET* transcript ratio observed among PDX models (Fig. 5G and fig. S9F).

### Ectopic overexpression of mutant *EGFR* is sufficient to confer MET inhibitor resistance and induce classic EGFR/MET codependency phenotype in MET-dependent models

**Fig. 6.**
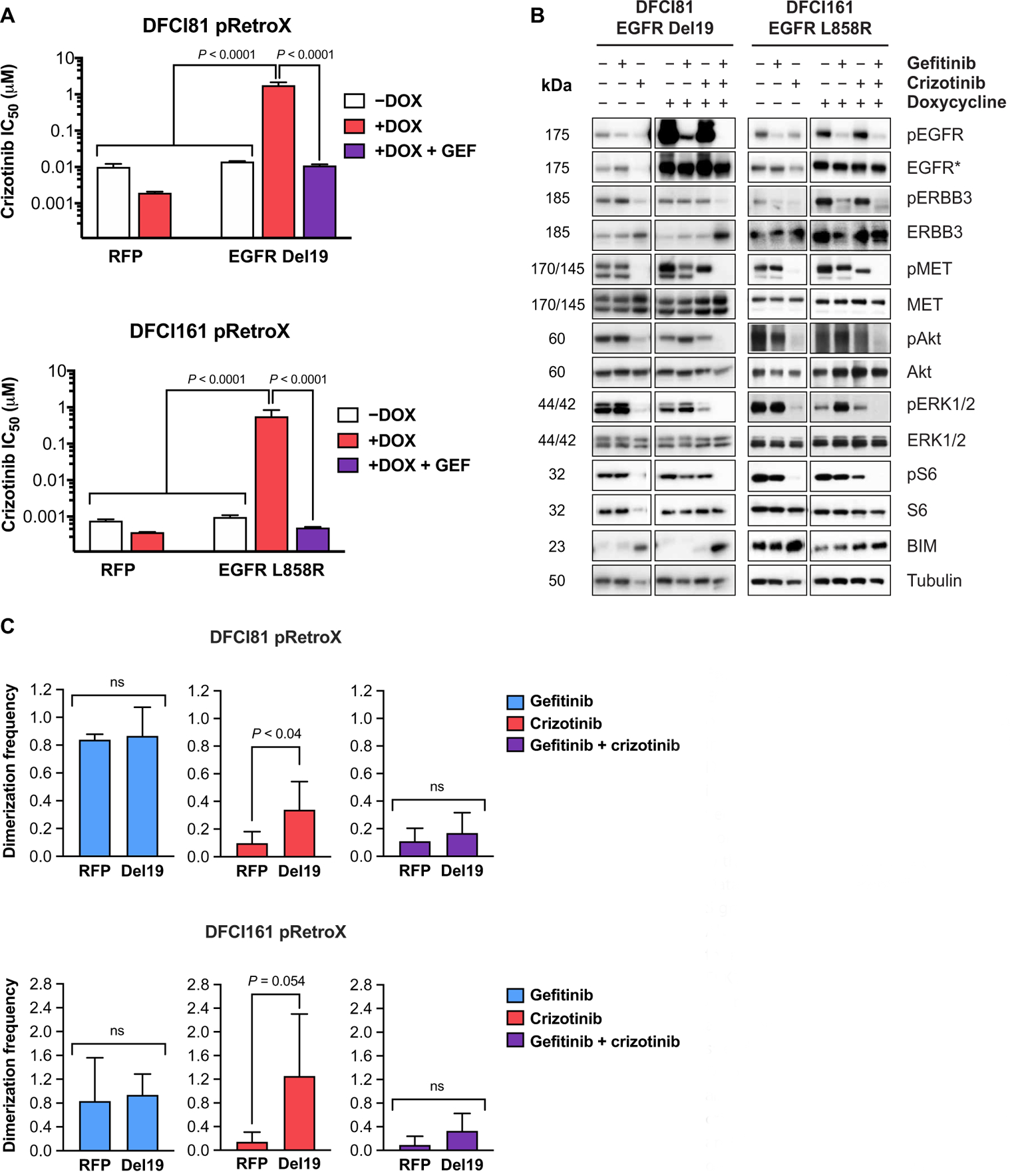
Effects of ectopic overexpression of mutant EGFR in DFCI81 and DFCI161 cell lines. (**A**) Quantification of drug sensitivities in the presence of doxycycline-inducible expression of EGFR Del19 and EGFR L858R in DFCI81 and DFCI161 cells, respectively. Drug sensitivity in the presence of a doxycycline-inducible control RFP vector is shown for comparison. IC_50_ values were derived from cell viability readout after a 96-hour drug treatment with a nine-point dose curve (*n* = 6 replicates per dose). Data shown are representative of three replicate studies. Significance of *P* < 0.0001 was determined by one-way ANOVA of technical replicates, followed by Tukey’s posttest for multiple comparisons. (**B**) Assessment of downstream ERK1/2 and Akt activation in the presence of doxycycline after treatment with single-agent and combined gefitinib and crizotinib. EGFR* denotes corresponding mutant-specific EGFR antibody (Del19 for DFCI81 and L858R for DFCI161). (**C**) Quantification of ERBB3-p85 dimerization after induction of ectopic mutant *EGFR* overexpression and crizotinib treatment. Bars on graphs represent means and SD of normalized quantification for three independent studies. Statistical significance for each treatment pair was assessed by *t* test.

Having observed that *EGFR*-mutant, MET-dependent models had reduced *EGFR:MET* transcript ratios compared to EGFR-dependent and codependent models, we set out to determine whether ectopically modulating the *EGFR:MET* expression ratio would shift drug sensitivity. Using a doxycycline-inducible expression vector, we transduced DFCI81 and DFCI161 cells with either activating mutant *EGFR* (corresponding to the activating mutation present in parental cells) or a red fluorescent protein (*RFP*)–encoding control vector. Although overexpressing mutant *EGFR* in a doxycycline-inducible manner in the DFCI81 and DFCI161 models had no effect on the single-agent gefitinib resistance of the cells (fig. S9G), it induced resistance to single-agent crizotinib and recapitulated EGFR/MET codependency in both cell lines (Fig. 6A). We further demonstrated doxycycline-mediated up-regulation of EGFR del19 (DFCI81) and EGFR L858R (DFCI161) (Fig. 6B and fig. S9H). Examination of downstream signaling revealed that ectopic overexpression of mutant *EGFR* in our MET-dependent cells not only phenocopied the drug resistance profile of EGFR/MET-codependent cells but also induced codependent regulation of downstream kinases. As we had previously observed in the HCC827GR6 cells, phosphorylation of ERBB3, ERK1/2 and Akt, and the downstream ribosomal protein S6 was only inhibited with the combination of crizotinib and gefitinib in mutant EGFR–overexpressing DFCI81 or DFCI161 cells (Fig. 6B). Last, we assessed the effects of ectopic mutant EGFR overexpression on the ERBB3-p85 interaction. When the EGFR-overexpressing DFCI81 and DFCI161 pRetroX cells were treated with EGFR and MET kinase inhibitors after doxycycline induction, we observed a shift of the ERBB3-p85 interaction from single-agent crizotinib sensitivity toward EGFR/MET codependence in both cell lines, defining the ERBB3-p85 interaction as a potential mediator and predictor of oncogene dependence in *EGFR*-mutant NSCLC models (Fig. 6C and fig. S9I). These observations highlight that oncogene dependence in *EGFR*-mutant NSCLC is determined not only by the presence of an activating mutation but also by the relative expression stoichiometry and functional protein interactions of the putative oncogenic driver (fig. S10).

## DISCUSSION

*MET* amplification in *EGFR*-mutant lung cancer was first described in an in vitro selected drug-resistant cell line (9) and subsequently established as a clinical marker of EGFR TKI resistance in patients (8, 10). Several reports of *MET* amplification–mediated resistance to targeted therapies have emerged thereafter, including to anaplastic lymphoma kinase (ALK) inhibitors in *ALK*-rearranged NSCLC, cetuximab in colorectal cancers, and antiangiogenic therapies in glioblastoma (40–42). With the rise of osimertinib as a first-line therapy for patients with *EGFR*-mutant NSCLC and ensuing reduction in the incidence of *EGFR* T790M–mediated resistance, the share of acquired resistance mediated by *MET* amplification has increased in patients with EGFR-driven tumors.

Traditionally, the presence of *MET* amplification has been clinically addressed through the addition of a MET inhibitor to the original targeted therapy, and the efficacy of combining EGFR and MET inhibitors in patients with concurrent *EGFR* mutation and *MET* amplification is firmly established (17, 18). However, two-drug combinations can lead to an increase in toxicity, necessitating treatment interruptions or dose reductions, which could limit therapeutic efficacy (43). We propose here that the biology of *MET*-amplified, *EGFR*-mutant NSCLC renders such combination regimens unnecessary in some cases.

Here, we established and characterized six patient-derived models that may represent a clinical subset of *EGFR*-mutant, EGFR TKI–resistant NSCLCs exhibiting sensitivity to MET inhibitor monotherapy. Because each of these cancers retains expression and activation of the oncogenic *EGFR* allele, loss of EGFR codependence in these models was not intuitive. However, we demonstrated that neither the genomic presence nor the sustained expression and phosphorylation of mutant EGFR predicted EGFR/MET codependence. Instead, all six of our *EGFR*-mutant, *MET*-amplified models showed downstream signaling pathways solely dependent on MET activation. Furthermore, there was no benefit, in vitro or in vivo, of adding an EGFR inhibitor to a MET inhibitor in any of the models studied.

Although there are several precedents of cancers evolving from oncogene dependence to independence [including small cell transformation in *EGFR*-mutant NSCLC and ABL kinase–independent imatinib resistance in chronic myelogenous leukemia (44, 45)], we characterize here a subset of cancer that exhibits a switch in dependence to another oncogene while retaining expression and activation of the original driver oncogene. Why, how, and when this switch from EGFR to MET dependence occurs are not clear. Our findings suggest that the relative expression of EGFR and MET dictates this dependency and that by modulating them, we can also modulate oncogene dependency and drug sensitivity. Understanding the minimum threshold required for this switch, the mechanistic basis—whether genomic or epigenetic—behind reduced mutant *EGFR* expression, and why and how at that juncture MET solely activates ERBB3 and all downstream signaling will require additional investigation.

*MET* amplification can exist as a de novo mechanism of resistance to EGFR inhibitors or arise through the selection of a preexisting *MET*-amplified subclone during drug treatment (11, 46). Likewise, single-agent MET dependence occurs both as a de novo and acquired resistance mechanism, highlighting the plasticity of *EGFR*-mutant lung cancers to rewire their signaling networks. We previously described an acquired *MET* D1228V mutation mediating resistance to osimertinib/savolitinib in DFCI307 (14). Development of savolitinib resistance through a *MET* mutation after an initial clinical response and subsequent sensitivity of the tumor to a structurally distinct MET TKI strongly suggest that the tumor retained dependence on MET signaling throughout the course of patient therapy (14).

It is interesting to speculate why our findings contrast with the current treatment paradigm for patients with *EGFR*-mutant, *MET*-amplified NSCLC. Our observations stemmed from xenografts generated directly from the tumors of patients who developed clinical resistance (de novo or acquired) to EGFR inhibitors, without prior in vitro propagation. In addition to PDXs, our models spanned a spectrum of modalities including patient-derived cell line and organoid models and a PDX-derived organoid model (DFCI81). Hence, our observations of *MET* dependence in *EGFR*-mutant lung cancers are unlikely to be an experimental artifact. In contrast, the HCC827GR6 cell line was selected in vitro to be EGFR inhibitor resistant. Whether this difference alone accounts for the distinct drug sensitivity profiles remains to be determined. It is likely that both MET dependency and EGFR/MET codependency exist in the clinic. Recent reports have described single-agent MET inhibitor sensitivity in *EGFR*-mutant patients, suggesting that a switch to MET dependence can occur clinically (47–49). However, it is presently impossible to ascertain the clinical prevalence of single-agent MET dependency in *EGFR*-mutant lung cancer, because genotyping of lung cancers is most often based on DNA sequencing, which would not capture diminished expression of the mutant *EGFR* allele. A limitation of the present work is that only one model, HCC827GR6, was identified to exhibit a high mutant *EGFR:MET* transcript ratio and EGFR/MET codependence. In our limited series of *EGFR*-mutant, *MET*-amplified patient specimens, the vast majority had similar small ratios of mutant *EGFR:MET* expression as our single-agent MET inhibitor–sensitive PDX models. These findings require confirmation in larger patient cohorts but raise the possibility that most patients whose tumors harbor concurrent *EGFR* mutation and *MET* amplification could be treated with a single-agent MET inhibitor, although definitive proof can only come from a dedicated clinical study. An additional limitation of our study is that the in vivo treatments were carried out over 28 days, a much shorter duration than a typical clinical treatment course. It is possible that prolonged treatment with a single-agent MET inhibitor in patients could lead to changes in the tumor or the tumor microenvironment, including changes in growth factor expression, necessitating treatment with a combination of an EGFR and MET inhibitor, although we did not observe this to be the case in the course of our studies.

An ability to predict MET dependence de novo would permit treatment of a subset of patients with MET inhibitor monotherapy rather than with a less well-tolerated EGFR/MET inhibitor combination regimen, particularly if there is no therapeutic benefit of adding an EGFR inhibitor. We have identified a low *EGFR:MET* expression ratio as a robust predictor of single-agent MET inhibitor sensitivity in all six of our primary models; these results warrant validation in larger clinical cohorts. Additional studies are needed to develop approaches for optimal identification of patients who would benefit from single-agent MET TKI treatment and to determine whether our observations extend to other cancers where *MET* amplification has been described as a drug resistance mechanism.

## MATERIALS AND METHODS

### Study design

The objective of this study was to understand the mechanisms underlying sensitivity to single-agent *MET* inhibition among patients with *EGFR*-mutant NSCLC using PDX, cell line, and organoid models. Models were established from six patients over the course of the research. All patient specimens and data were collected in accordance with the Declaration of Helsinki and approved by the Dana-Farber Cancer Institute Institutional Review Board. Patients included in the study provided written informed consent, and all laboratory models derived from patient tissues were deidentified to protect patient privacy. All in vivo studies were randomized but unblinded, and replication was determined by the format and scope of each experiment, as described below.

### PDX models

All in vivo studies completed at Dana-Farber Cancer Institute were conducted in strict accordance with Dana-Farber Animal Care and Use Committee guidelines. Briefly, NSG (NOD.Cg-Prkdc^scid^ Il2rg^tm1WjI^/SzJ) mice were implanted with expanded primary cells or tumors, as previously described (50). PDX tumors for DFCI161, DFCI202, and MR007 (LCx-MR007PD-AR) were derived from pleural effusions collected from patients as part of routine clinical care. Effusions were immune depleted and enriched for cancer cells, and these cancer cells were cultured on plastic for 3 days in RPMI 1640 media supplemented with 10% fetal bovine serum (FBS) and 1% antibiotic before subcutaneous implantation. The PDX tumor for DFCI307 was derived from a surgical biopsy and implanted directly into the subrenal capsule of mice for expansion. After initial implantation, the DFCI161, DFCI202, MR007, and DFCI307 PDX models were expanded and passaged continually in mice as subcutaneous tumors, without touching plastic. All tumors used in PDX efficacy and pharmacodynamic studies were implanted subcutaneously. For the DFCI81 tumor, a patient-derived cell line was established and used for growing the subcutaneous xenograft tumor model. DFCI81 cells grown in RPMI 1640 media supplemented with 10% FBS and 1% antibiotic were harvested, and 5 × 10^6^ cells per mouse in 30% Matrigel (Corning) were implanted subcutaneously in mice. All PDX tumors and DFCI81 cells were implanted in 8- to 10-week-old female NSG mice purchased from the Jackson Laboratories (005557-NSG; RRID: IMSR_JAX:005557). After implantation, tumor establishment and growth were monitored by caliper measurements twice per week. Sample sizes of tumor-implanted mice were calculated to minimize the number of animals used, based on predetermined tumor take rates, variations among PDX growth kinetics, and number of treatment arms for each study. When patient- and cell line–derived PDX tumors reached 100 to 200 mm^3^ (with sizes varying based on PDX model and tumor growth kinetics), mice were randomized by tumor volume into various treatment groups. Mice harboring PDX tumors for DFCI81, DFCI161, and DFCI202 were treated with crizotinib hydrochloride (100 mg/kg) (DFCI81 and DFCI202 studies, MedChemExpress; DFCI161 study, Aurum Pharmatech) and with erlotinib hydrochloride (30 to 50 mg/kg) (DFCI81 and DFCI202 studies, Selleck; DFCI161 study, crushed Tarceva tablets). Mice harboring patient-derived DFCI307 and MR007 xenograft tumors were treated with 12.5 and 15 mg/kg, respectively, of savolitinib and with 25 and 10 mg/kg, respectively, of osimertinib, provided by AstraZeneca. Vehicle control mice were treated orally with either 0.5% hydroxypropyl methylcellulose (HPMC, Sigma-Aldrich) (DFCI161 and DFCI307) or 6% Captisol (CyDex Pharmaceuticals Inc.) (DFCI81 and DFCI202) in autoclaved water, depending on the drug formulations for each study. Mice were dosed once daily by oral gavage, and tumor measurements were taken using calipers twice a week, as previously described (46). At the time of each measurement, mice were weighed to affirm that treatment regimens were well tolerated. Mice exhibiting a 15% body weight loss were given a drug holiday until body weights recovered. Animals were euthanized if the tumor volume exceeded 2000 mm^3^ or if the tumors became ulcerated/necrotic, and tumor samples were harvested and snap-frozen for subsequent analysis. Animal studies completed at the Dana-Farber Cancer Institute were conducted with the approval of the Institutional Animal Care and Use Committee in an American Association for Accreditation of Laboratory Animal Care (AAALAC)–accredited vivarium and in accordance with the National Institutes of Health Guide for the Care and Use of Laboratory Animals. For sequencing and pharmacodynamic studies, mice with 200 to 400 mm^3^ of tumor volumes were dosed for 3 days with kinase inhibitors or vehicle control, and tumors were harvested and snap-frozen in liquid nitrogen for analysis. Tumor tissues used for protein and RNA extraction were first homogenized using FastPrep-24 (MP Biomedicals) in corresponding lysis buffers per manual instructions.

### Primary cell line establishment and maintenance

DFCI81 and DFCI161 cell lines were established from pleural effusions using previously described methods (50). After establishment, both cell lines were maintained in RPMI 1640 media supplemented with 10% FBS and 1% penicillin/streptomycin (R10). DFCI649 organoids were established from pleural effusion and cultured in Renaissance Essential Tumor Medium supplemented with 5% FBS, 1% penicillin/streptomycin, and B-27. Sources, culture parameters for other cell lines, and additional details are in Supplementary Materials and Methods. Cultured cells regularly tested negative for mycoplasma.

### Statistical analysis

Experimental data shown represent the outcomes of at least three biological replicate studies, as specified in the figure legends. Where applicable, statistical significance was determined by analysis of variance (ANOVA) or *t* test, with the parametric test criteria of normal distribution and homogeneity of variance verified by Shapiro-Wilk’s and Levene’s tests, respectively. A *P* value threshold of <0.05 was considered statistically significant, with *P* values specific to experiments and ANOVA posttests applied for multiple comparisons specified in the corresponding figures and figure legends.

## Supplementary Material

Supp_Data_S1

Supplement

## Data Availability

All data associated with this study are present in the paper or the Supplementary Materials. All materials are available through a material transfer agreement. The MR007 (LcxMR007PD) model is available for studies through collaboration with XenTech (Evry, France).
