# Supplementary material for: Oncogenic switch and single-agent MET inhibitor sensitivity in a subset of EGFR-mutant lung cancer
Source: Sci Transl Med. Author manuscript; Available in PMC 2021 Nov 28. (PMC8627689; doi:10.1126/scitranslmed.abb3738)
Supplement: Supplement [file NIHMS1758040-supplement-Supplement.pdf]

Science Translational Medicine
AAAS

Supplementary Materials for

Oncogenic switch and single-agent MET inhibitor sensitivity in a subset of *EGFR*-mutant lung cancer

Pınar Özden Eser *et al.*

Corresponding author: Pasi A. Jänne, pasi_janne@dfci.harvard.edu

Sci. Transl. Med. **13**, eabb3738 (2021)
DOI: 10.1126/scitranslmed.abb3738

The PDF file includes:

Materials and Methods
Figs. S1 to S10
Tables S1 to S3

Other Supplementary Material for this manuscript includes the following:

Data file S1

Supplementary Materials

Cell line maintenance

PC9 cells (RRID: CVCL_B260) were a generous gift from Dr. Nishio (Kinki University, Osaka, Japan; 2005); H1975 (CRL5908; RRID: CVCL_1511), H3255 (CRL2882; RRID: CVCL_6831), HCC827 (CRL2868; RRID: CVCL_2063), and H1993 (CRL5909; RRID: CVCL_1512) cells were purchased from ATCC; EBC-1 cells were purchased from the JCRB Cell Bank (JCRB0820; RRID: CVCL_2891); and HCC827GR6 (RRID: CVCL_V623) cells were derived through prolonged gefitinib selection of HCC827 parental cells *(10)*. H3255 cells were maintained in ACL-4 media (Fisher Scientific) supplemented with 10% fetal bovine serum (Sigma Aldric) and 1% antibiotic (Life Technologies), while all remaining lung cancer cell lines were maintained in complete RPMI-1640 (Life Technologies) media (supplemented with 10% FBS and 1% antibiotic, as above). HEK293T/17 cells were obtained from ATCC (CRL-11268; RRID: CVCL_1926) and maintained in complete DMEM media (Life Technologies). DFCI81 and DFCI161 transduced with doxycycline-inducible constructs were cultured and assayed exclusively in RPMI-1640 media supplemented with tetracycline-free, FBS (Takara Bio). All cell lines were used within 40 passages of their acquisition. Adherent cells were detached from culture flasks for passaging and plating through trypsinization (Trypsin: Life Technologies) following a wash in phosphate-buffered saline (PBS: Life Technologies) to remove residual FBS. All patient-derived and established cell lines were routinely tested for mycoplasma using the Mycoplasma Plus PCR Primer Set (Agilent).

Patient-derived organoid model establishment and culture

Pleural effusion specimens were obtained as part of clinical care from patients resistant to EGFR TKI treatment. After red blood cell lysis (RBC Lysis Buffer: Boston BioProducts) and CD45 depletion by MicroBeads (Miltenyl Biotex), ice-cold Matrigel (Corning) was added to cells and incubated on pre-warmed 6 cm plates to solidify. To generate primary cancer organoids, cells were cultured in Renaissance Essential Tumor Medium (RETM: Cellaria Biosciences) supplemented with B-27 Serum-Free Supplement (Thermo Fisher Scientific). Organoids passaged over three times were used for experiments or cryopreserved.

<u>Cell growth, viability, and apoptosis assays</u>

Cell viability assays were used to quantify drug sensitivity, as previously described. IC_{50} values were extrapolated from toxicity curves and displayed using GraphPad Prism Software. Caspase 3/7 activity was monitored and quantified using IncuCyte imaging technology, as previously described *(48)*. Briefly, cells were seeded at a density of 3,000-5,000 cells/well in 96-well plates (Westnet Inc. or Fisher Scientific). For drug sensitivity assays in the presence of growth factors, cells were seeded in complete RPMI-1650 media supplemented with a final concentration of 10 ng/mL of recombinant growth factor, or a corresponding volume of vehicle 0.1% (m/v) BSA (diluted in ultrapure water and filter sterilized) (**table S3**). Cells transduced with doxycycline-inducible constructs were seeded in complete RPMI-1640 media with a final concentration 2.5 µg/mL doxycycline hyclate (Sigma Aldrich), or a corresponding volume of vehicle control water. The day following seeding, kinase inhibitors (**table S3**) were administered at nine doses at equal intervals ranging from 1 nM to 10 µM, with each concentration set up in six wells, representing technical replicates. Following 96-hour incubation, cells were washed once in PBS, and their viability was quantified using MTS assay (Promega) or CellTiter-Glo (Promega),

adhering to manufacturers' instructions. MTS Reagent Powder reconstituted to 0.5 g/L in DPBS (Life Technologies) was diluted further 1:5 in serum- and antibiotic-free RPMI-1640 and supplemented with 1-4% phenazine methosulfate (PMS: Sigma Aldrich). Following addition of complete MTS reagent, cells were incubated for 4-8 hours (or as long as it took for the MTS solution to turn dark brown) in a humidified CO_2 incubator at 37°C. Absorbance was quantified on a PolarSTAR Omega Plate Reader (BMG Labtech). CellTiter-Glo working solution was 1:4 diluted in Opti-MEM Reduced Serum Medium (Life Technologies) and added to cells cultured in white 96-well plates and incubated at room temperature for 10 minutes on an orbital shaker. Luminescence was quantified by plate reader. All absorbance or luminescence values were normalized to corresponding untreated control wells on each plate, and IC_{50} values were extrapolated using GraphPad Prism non-linear regression analysis. Since the highest drug concentration cells were exposed to was 10 µM of drug, all IC_{50} values that appeared to exceed this dose were denoted as 10 µM. Unless otherwise noted, each experiment shows representative data from at least three biological replicates, with means, error bars, and statistical significance assessed from the technical replicates of a single experiment.

Cells for drug concentration matrix studies were seeded at 1,000 cells-well in white 384-well plates (Corning), avoiding plate edges to prevent evaporation. Inhibitor dose gradients were added the following day using the HP D300e Digital Dispenser, and cells were incubated for 96-hours. Viability was quantified using the CellTiter-Glo, according to manufacturer's protocols, and analyzed as described above. Where applicable, Bliss synergy scores were calculated using the SynergyFinder web application *(23)*. For the crystal violet readout, cells were seeded at 10,000 – 40,000 cells/well, depending on cell growth parameters, in 48-well plates, and treated the following day with corresponding drug concentration gradients. Following four-day

incubation in drug, cells were fixed in 10% formalin, and stained with 0.05% (w/v) crystal violet diluted in ultrapure water containing a final concentration 20% ethanol v/v. Following up to one hour of staining, wells were rinsed twice in ultrapure water, and placed upside-down to dry completely prior to imaging.

For active caspase 3/7 quantification, cells were seeded at 3,000-5,000 cells/well and treated the following day with TKIs and CellEvent Caspase-3/7 Green ReadyProbes reagent (Fisher Scientific). The confluence and caspase activation of cells were monitored using an IncuCyte FLR Live Cell Analysis system (Essen Bioscience). At the conclusion of the study, data were analyzed using IncuCyte image analysis software. Caspase-3/7-positive fluorescent signal count was normalized to confluence following 96 hours of TKI treatment.

Organoid Cell Viability Assay

The cells in 3D matrices were harvested and dissociated using TrypLE Express Enzyme (Thermo Fisher Scientific) at 37°C. The single cells were resuspended in cold RETM/10% Matrigel and plated into 384-well ultra-low attachment microplates (Corning) at 1000 cells per well. On the next day, cells were treated with DMSO, osimertinib and crizotinib dose gradients, or 500 nM osimertinib combined with crizotinib dose curve, as indicated. Each dose had more than three replicates. At the study endpoint, the representative images of each condition were captured under microscope (Olympus CKX41 with DP72 camera). The viability assay was performed using CellTiter-Glo 3D (Promega) according to the manufacturer’s protocol. Statistical significance was accessed using ANOVA and Turkey’s post-test for multiple comparisons.

Co-immunoprecipitation and Western blotting

Cells were seeded at 500,000 – 750,000 cells/100 mm dish for Western blots, and 1.5-2 x 10^6 cells/150 mm dish for immunoprecipitation. Cells were treated the following day with drugs, concentrations, and durations specific to each study, and were lysed in RIPA Buffer or NP-40 Lysis Buffer (**table S3**). Lysates were quantified, diluted, and immunoprecipitated using previously described protocols (9). Briefly, for western blot, cells were lysed in RIPA buffer with Triton X (Boston BioProducts Inc.). Cells for immunoprecipitation and tumors for western blot were lysed and homogenized, as necessary, in 1X NP-40 buffer (Boston BioProducts Inc.). Lysis buffers were supplemented with phosphatase inhibitor (Sigma Aldrich) and protease inhibitor tablets (Sigma Aldrich). Lysates were quantified by BCA assay (Thermo Fisher Scientific) and measured on a PolarSTAR Omega Plate Reader. Lysates were normalized according to a BCA standard curve run alongside samples. For Western blots, samples were diluted in SDS sample reducing buffer (Boston BioProducts Inc.), heated to 100°C, and stored at -80°C until analysis. For CO-IP, quantified lysates were uniformly diluted in NP-40 buffer to 300 μg – 1 mg per IP in 250 – 500 μL volume per IP in 1.7 mL microcentrifuge tubes (Thomas Scientific). Corresponding antibodies (**table S3**) were added at a 1:50 or 1:100 dilution, depending on product recommendations, and tubes were incubated overnight at 4°C on a rotator. The following day, antibodies and attached proteins were incubated with A/G-Plus Agarose beads (Santa Cruz Biotechnology) for >1 hour at 4°C, and subsequently immunoprecipitated by centrifugation at 500xg for 5 minutes. Beads were washed three times in 1X NP-40 buffer prior to resuspension and boiling in reducing buffer. As with western blot lysates, samples were stored at -80°C until analysis following the addition of reducing buffer.

Samples for Western blot ands immunoprecipitation analysis were electrophoresed alongside a protein size standard ladder (Bio-Rad Laboratories) on precast 4-12% Bis-Tris gels

(Life Technologies) and transferred to Immobulin-P membranes (Fisher Scientific), following established protocols *(46)* and using the reagents detailed in **table S3**. After protein transfer, Immobulin-P membranes were cut by size, blocked with 5% (m/v) non-fat milk (Bio-Rad Laboratories) in 1X Tris-Buffered Saline (Westnet Inc.)/0.1% Tween®20 (Sigma Aldrich), and incubated overnight on a rocker at 4°C in primary antibodies (**table S3**) diluted 1:1000 in 3% (m/v) BSA in TBS-T (1X Tris-Buffered Salinen/0.1% Tween20). The following day, membranes were washed in TBS-T, incubated for one hour at room temperature in HRP-conjugated secondary antibody diluted 1:5000 in 3% BSA (**table S3**), re-washed, and developed with HRP substrate (Life Technologies). Western blots were imaged and analyzed on an Amersham Imager 600 (GE Healthcare Life Sciences), as previously described *(46)*. Immunoprecipitation studies were quantified using ImageQuant TL Image Analysis Software (GE Healthcare Life Sciences). Uniform grids were used to ensure that an equal region was quantified for each cell line/protein. For ERBB3 pulldowns, p85 bands were quantified and normalized to ERBB3 bands from same co-IP; ERBB3 quantification was normalized to p85 in reciprocal pulldown studies. Where applicable, Western blots were quantified using adobe photoshop following image inversion and selection of uniform areas. All quantified Western blot bands were normalized to coinciding loading control or coinciding loading control and total protein, as indicated in figure legends, prior to plotting.

qRT-PCR, and NGS

RNA was extracted from cell lines and snap-frozen tumors using TRIzol or the Qiagen RNeasy RNA Isolation kit, abiding by manufacturers' protocols. cDNA was synthesized from RNA using the QuantiTect Reverse Transcription Kit from Qiagen, as previously described *(48)*. Following

cDNA synthesis, real-time qPCR was carried out according to manufacturers' instructions using TaqMan Gene Expression Master Mix from Applied Biosystems (Life Technologies), and primers for human EGFR (FAM reporter) and MET (Fam reporter). Gene expression was normalized to β-actin (VIC reporter) and GUSB (VIC reporter) measurements taken from each well (**table S1**). qRT-PCR was carried out in optical 96-well plates (Fisher Scientific) on a StepOne Real-Time PCR System (Applied Biosystems), and three technical replicates were measured for each cell line/gene. Each cell line and xenograft PCR quantification study was repeated for a total of at least three biological replicates, unless otherwise noted. Where relevant, EGFR:MET expression ratios were calculated as $2^{-(\Delta Ct(EGFR)- \Delta Ct(MET))}$, where $\Delta Ct = Ct_{EGFR \text{ or } MET} - Ct_{ACTB \text{ or } GUSB}$. Data were visualized and statistical analyses were carried out using GraphPad Prism software. To determine allelic expression fraction, exons 19 and 21 of EGFR were amplified from cDNAs using Phusion High-Fidelity Polymerase (New England Biolabs) and cDNA specific primers (**table S1**) in accordance with manufacturer protocols and submitted to the Massachusetts General Hospital Center for Computational and Integrative Biology DNA core for targeted next-generation sequencing.

DNA FISH and BaseScope RNA ISH Analysis

MET genomic FISH was carried out on 10% formalin fixed cell lines or formalin fixed paraffin embedded (FFPE) tumor tissues with centromere 7 (CEN7) as a control marker, using previously published protocols and probes *(48)*. Mean *MET* copy number (CN) was based on analysis of at least 30 nuclei per specimen. *MET* amplification was defined as either MET-CN > 5 or MET/CEP7 > 2, in accordance with the criteria used for clinical trial enrollment for osimertinib and savolitinib *(17)*. For BaseScope analysis, cell lines were pelleted prior to formalin fixation.

Cell line and tumor specimens were prepared by FFPE and arranged into tissue microarrays for submission to ACD Bio for processing and image scanning. BaseScope images were quantified as signal area/cell area using ImageJ image processing software.

RT-ddPCR

cDNA-specific RT-ddPCR primers were designed to span exon-intron junctions to exclude genomic DNA. Probes were designed with HEX or FAM reporter dye and ZEN/Iowa Black FQ quencher (**table S1**). Primers and probes were designed specific to wild type *MET* or mutant *EGFR*, with mutation-specific probes used for distinct *EGFR* exon 19 deletions. Primers and probes were manufactured by Integrated DNA Technologies. RT and ddPCR reactions were prepared using One-Step RT-ddPCR Advanced Kit for Probes in a reaction (20 µL) including 1xSupermix, 20U/µL reverse transcriptase, 15mM DTT, 900 nM primers, 250 nM probes and 10 ng RNA. After droplets generation (Automated Droplet Generator, Bio-Rad), the reactions cycled through following conditions: 42°C/60 min; 95°C/10 min; 40 cycles of 95°C/30 sec followed by 55°C/1 min; 98°C/ 10 min; and 4°C hold. The thermocycler plate was read by a QX200 Reader (Bio-Rad) and data analyzed by QuantaSoft software.

Expression Vectors and Viral Transduction

EGFR L858R and *EGFR* Del19 ORFs were cloned into the pRetroX doxycycline inducible plasmid by gateway cloning, as described in Supplementary Methods. Plasmids were validated by sequencing and transfected into HEK293T/17 cells alongside pRetro-gag/pol and MDM2.G retroviral plasmids (**table S1**). The doxycycline-inducible Retro-X Tet-One system acquired from Clontech Laboratories, Inc. was cloned in accordance with manufacturer's user manual.

EGFR Del19 and EGFR L858R open reading frames were PCR amplified from a donor vector using the primers described in **table S1**, and cloned into pRetroX-TetOne vector using the In-Fusion HD Cloning Plus Kit (Takara Bio), abiding by manufacturer protocols. Plasmids were transformed and grown in competent bacteria (Fisher Scientific), and isolated by plasmid miniprep (**table S3**). Following validation of cloned pRetroX-TetOne vectors by sequencing (**table S1**), EGFR expression plasmids were combined with Retro gag/pol packaging vector (Addgene) and envelope vector pMD2.G (Addgene), and transfected into HEK293/17 cells plated the previous day in complete DMEM media supplemented with tetracycline-free FBS. Transfection to make virus was carried out using the Xfect transfection reagent (Takara Bio) in accordance kit protocols, and complete DMEM/tetracycline-free FBS media was replenished less than 12 hours after transfection, and every 24 hours subsequently. Media containing 36-72-hour retrovirus was harvested from the supernatant of HEK293T cells, passed through a 0.45 μm syringe filter (BD and Fisher Scientific), supplemented with a final concentration of 10 μg/mL Polybrene (Santa Cruz Biotechnology), and added to DFCI81 and DFCI161. Transduction was carried out overnight, and virus-containing media was removed from cells within 24 hours. Following a two-day recovery period in complete RPMI-1640 supplemented with tetracycline-free FBS, transduced DFCI81 and DFCI161 cells were selected for two weeks with 2 μg/mL puromycin (Fisher Scientific), according to standard protocols *(46)*. DFCI81 cells ectopically overexpressing ERBB3 on a retroviral JP1540 vector or control empty vector plasmid (“FLAG”) *(33)* were generated and selected through an identical workflow as the pRetroX cells. All assays were performed following one week of puromycin washout, and within one month of transduction.

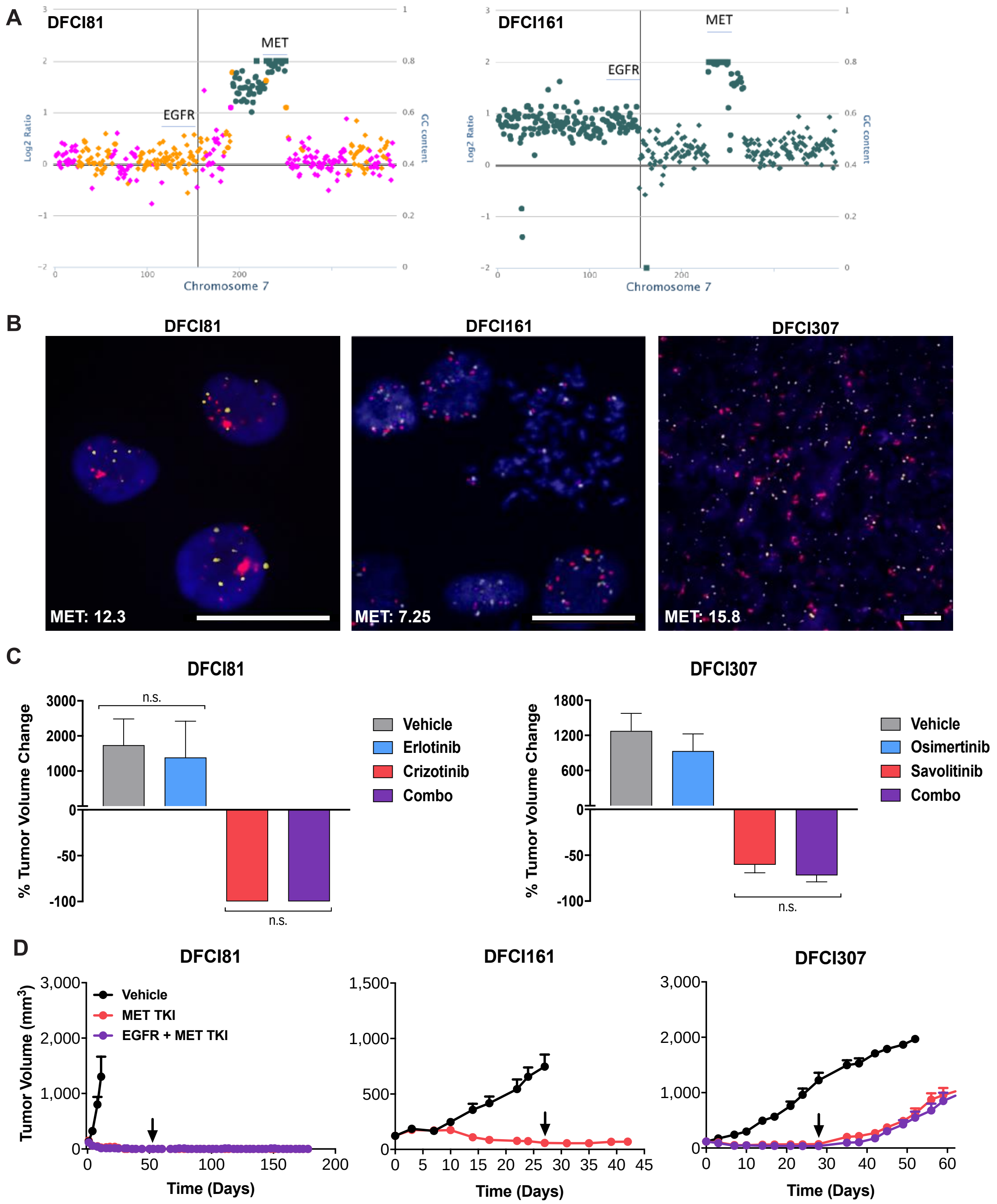

Figure S1: *MET* amplification and single-agent MET inhibitor response in patient-derived primary NSCLC models.

(A) Chromosome 7 oncoplots from patient-derived samples reveal *MET* copy number gain independent of the centromeric region in DFCI81 and DFCI161 cell line models. (**B)** FISH assay for genomic MET (red) and centromere 7 (CEN7, yellow) of DFCI81, DFCI161, and DFCI307 patient-derived cells reveals genomic MET amplification. The inset MET value for each FISH panel represents the mean MET copy number per cell. Each scale bar indicates a length of 20 µm. Images were captured using a Nikon Eclipse 80i Fluorescence Microscope. **(C)** The magnitude of single-agent MET inhibitor response in *EGFR*-mutant, MET dependent PDX models was statistically indistinguishable from combination treatment EGFR and MET inhibitors in *EGFR*-mutant, MET-driven DFCI81 and DFCI307 PDX models. Results from the waterfall plots shown in panel 1D were pooled, and a lack of statistically significant difference between treatment arms was determined by one-way ANOVA followed by Tukey's post-test for multiple comparisons. **(D)** The duration of single-agent MET inhibitor response following drug withdrawal (indicated by the arrows) in *EGFR*-mutant, MET dependent lung cancer models. Tumor outgrowth was measured during short-term and long-term drug withdrawal after single-agent MET inhibitor versus combined EGFR and MET inhibitor treatment.

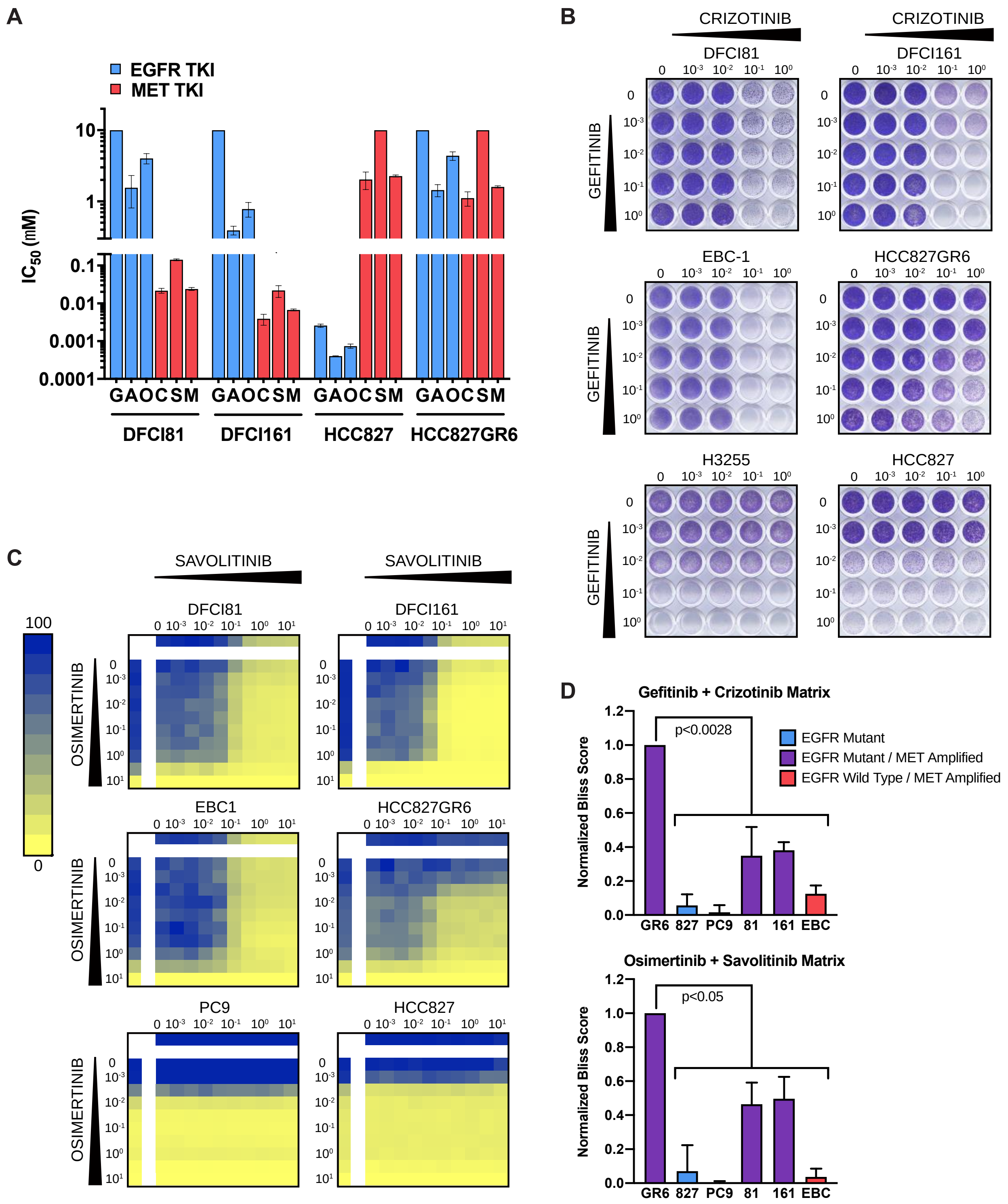

Figure S2: Single-agent and combination treatment of *EGFR*-mutant, MET-dependent models and controls with a panel of EGFR and MET kinase inhibitors.

(A) Comparison of drug sensitivities of EGFR-dependent HCC827 cells and EGFR/MET-co-dependent HCC827GR6 cells to MET-dependent DFCI81 and DFCI161 cells after treatment with a panel of EGFR inhibitors including gefitinib (G), afatinib (A), and osimertinib (O), and a panel of MET inhibitors including crizotinib (C), savolitinib (S), and merestinib (M). Each bar represents mean and standard deviation among technical replicates from a representative study. **(B)** Crystal violet staining reveals drug sensitivity to single-agent and combination TKI treatment following exposure to dose gradients of EGFR and MET inhibitors. Visualization of cell viability for *EGFR*-mutant, MET-dependent DFCI81 and DFCI161 cell lines and *EGFR* wild type MET-dependent EBC-1 cells compared to the EGFR/MET co-dependent HCC827GR6 model and EGFR-dependent H3255 and HCC827 cell lines. Doses shown are in µM. **(C)** Drug concentration matrices showing percent viability (0-100%) measured following treatment with an inhibitor dose gradient reveal the effects of the EGFR inhibitor osimertinib and the MET inhibitor savolitinib, as single-agents and combined at various doses, on EGFR/MET co-dependent HCC827GR6 cells compared to EGFR-mutant DFCI81 and DFCI161 cells. Drug sensitivities of MET-dependent EBC-1 cells and EGFR-dependent parental HCC827 and PC9 cells are shown for comparison. Cell viability was assayed following 96-hour drug incubation, and doses shown are in µM. **(D)** Bliss synergy scores were calculated from drug concentration matrices as previously described *(23)* and normalized to the score obtained for HCC827GR6. Statistical analysis by one-way ANOVA followed by Dunnett's multiple comparisons test revealed that the EGFR/MET co-dependent HCC827GR6 cells exhibited a significantly higher Bliss synergy score compared to EGFR-dependent and MET-dependent models.

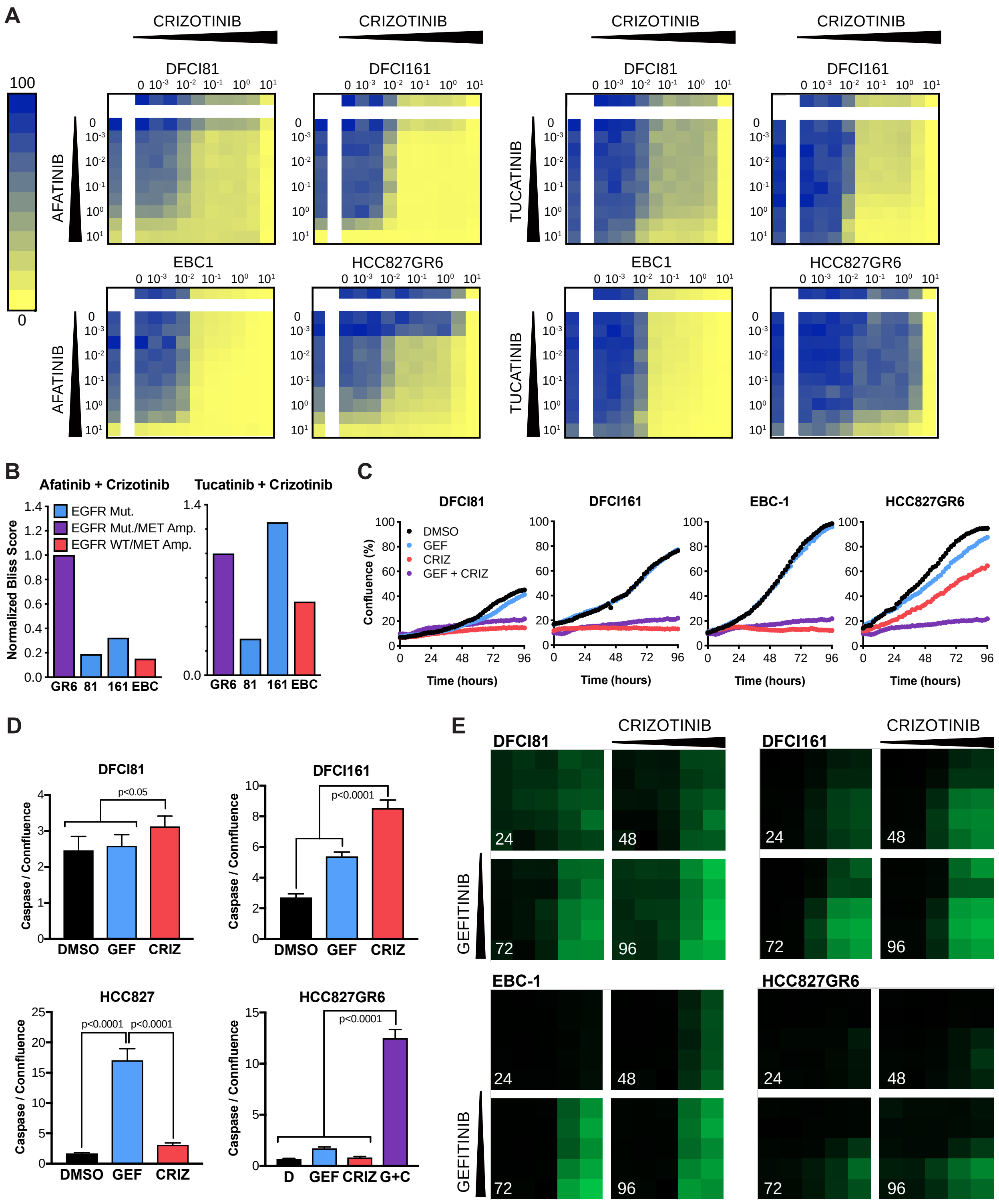

Figure S3: Resistance of *EGFR*-mutant, MET-dependent models to ERBB inhibition and quantification of drug-induced apoptosis.

(A) Drug concentration matrices showing percent viability (0-100%), as measured following 96-hour incubation with inhibitor dose gradients in the *EGFR*-mutant, MET-dependent DFCI81 and DFCI161 cell lines and *EGFR* wild type, MET-dependent control cell line EBC-1 compared to the EGFR/MET co-dependent HCC827GR6 cell line. Cell viability was quantified after treatment with the pan-ERBB inhibitor afatinib or the ERBB2 inhibitor tucatinib, administered alone or in combination with the MET inhibitor crizotinib. **(B)** Bliss synergy scores for the combination of crizotinib and afatinib or crizotinib and tucatinib were calculated as previously described *(23)* and normalized to the HCC827GR6 control. **(C)** Quantification of confluence over 96 hours of incubation in 1 μM of kinase inhibitors for DFCI81, DFCI161, EBC-1, and HCC827GR6 cell lines. **(D)** Quantification of caspase 3/7 activation following 96 hours of TKI treatment reveals apoptosis induction in DFCI81 and DFCI161 cells following gefitinib, crizotinib, or combination treatment. Data display representative mean and standard deviation among technical replicates. Statistical significance was assessed by ANOVA analysis followed by Tukey's multiple comparisons post-test. **(E)** DFCI81, DFCI161, EBC-1, and HCC827GR6 cell lines were treated across concentration gradients of the MET inhibitor crizotinib (dose escalation left-right, with concentrations of 0, 0.001, 0.01, 0.1, and 1 μM) and the EGFR inhibitor gefitinib (dose-escalation top-to-bottom, treated at the same contrations as crizotinib) and quantified for fluorescent caspase induction and confluence. The dosing matrices represent confluence-normalized capase activity at four time points taken at 24-hour increments, with black corresponding to no caspase activity and green representing maximal activity. Because the dynamic range of caspase activation varies by cell line, each heatmap was internally normalized to capture drug-induced caspase activity for the cell line of interest.

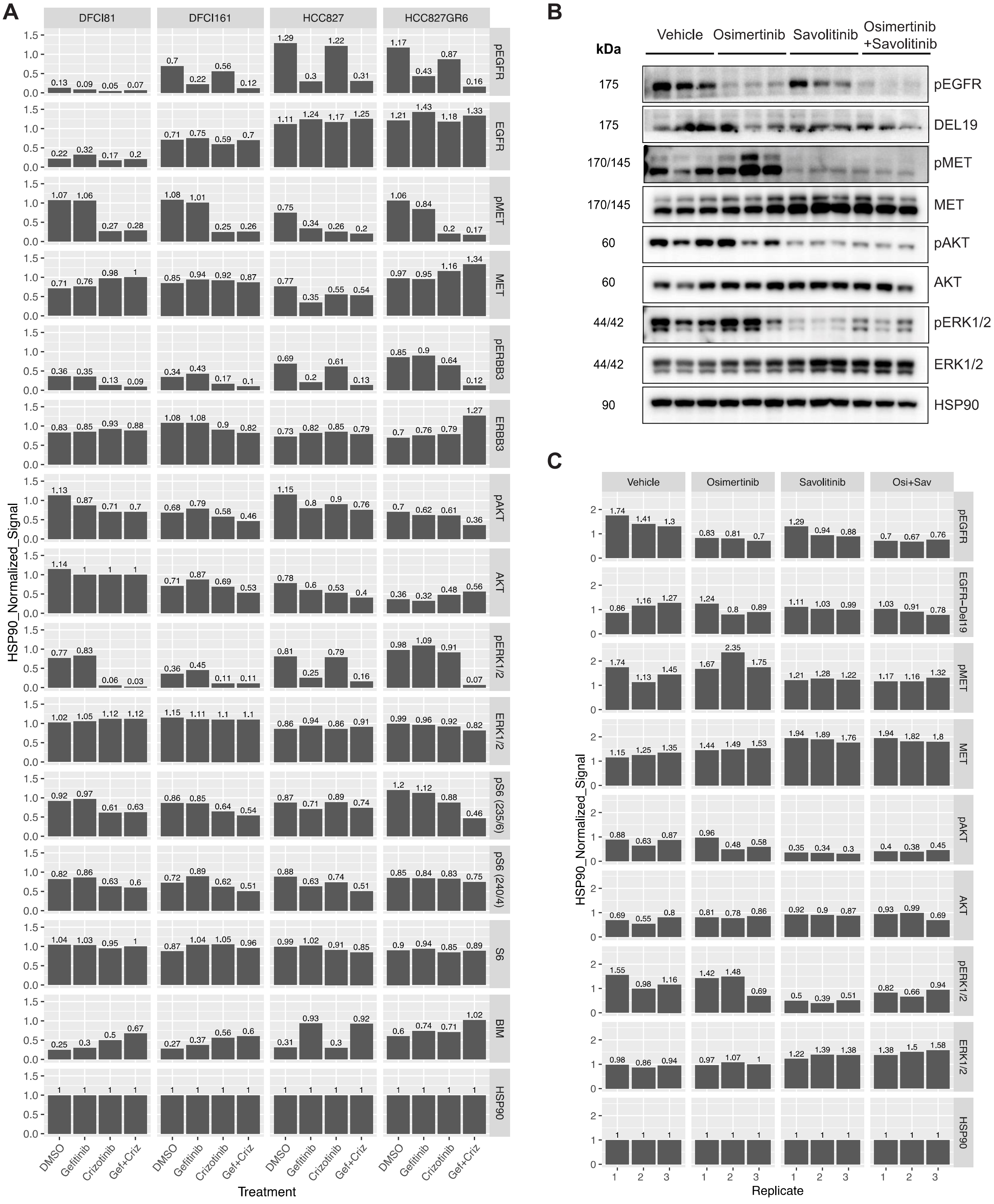

Figure S4: Quantification of Western blots following treatment of MET-dependent cell line and PDX models with EGFR and MET TKI.

(A) HSP90 loading control-normalized quantification of Western blot in Fig. 2D. **(B)** Activation of downstream signaling cascades following treatment of the DFCI307 patient-derived xenograft model with single-agent osimertinib, single-agent savolitinib, or combined osimertinib and savolitinib. **(C)** HSP90 loading control-normalized quantification of western blot in panel B.

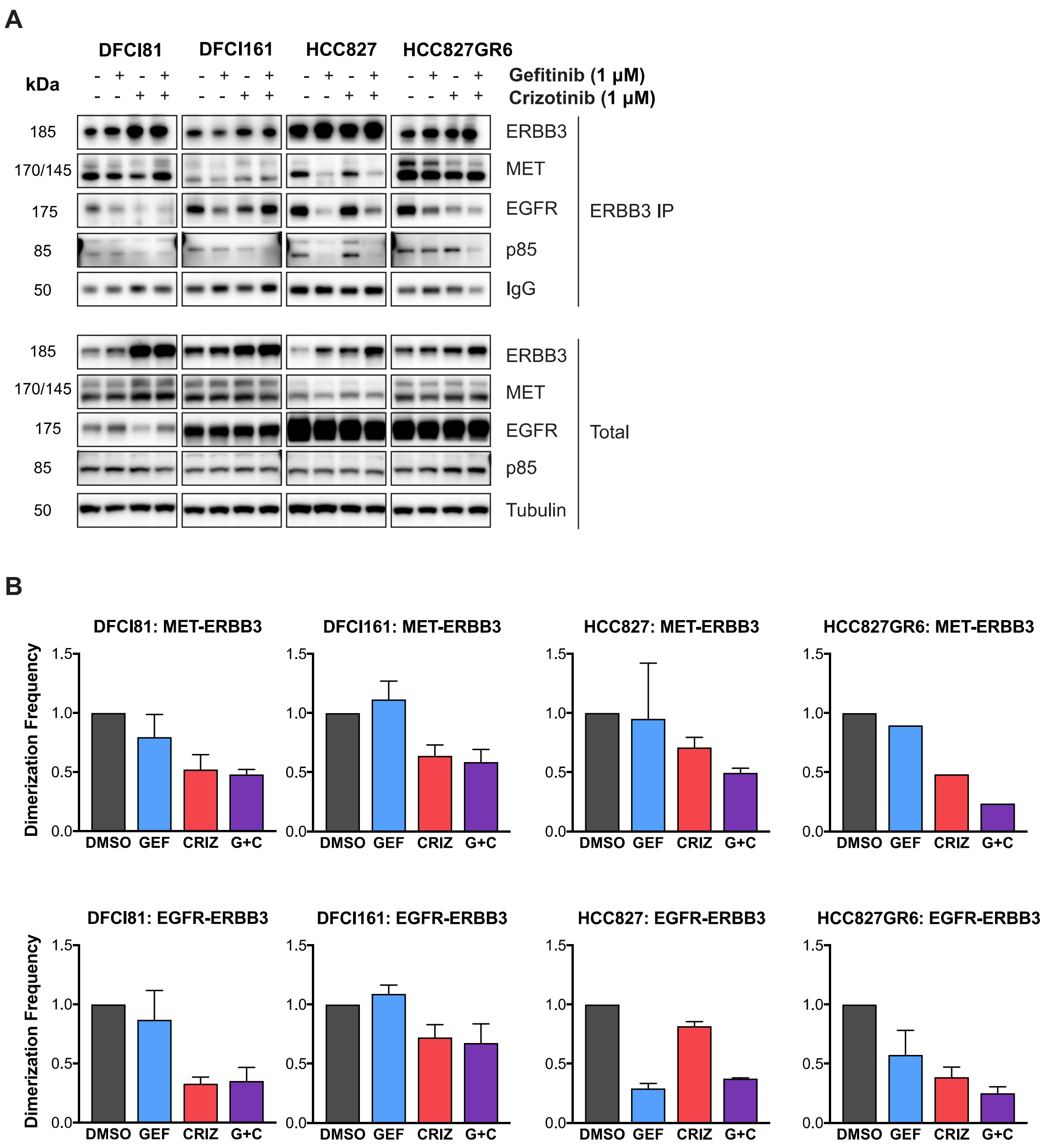

Figure S5: MET-mediated signaling and dimerization in DFCI81, and DFCI161 cell lines. **(A)** Co-immunoprecipitation studies to assess the impact of single-agent gefitinib, single-agent crizotinib, or combination TKI treatment on the interaction between MET kinase and ERBB3 in DFCI81 and DFCI161 cells. Cells were lysed for immunoprecipitation following 8 hours of treatment with TKI, and the Western blot is representative of biological replicate studies. **(B)**

Normalized quantification of MET-ERBB3 and EGFR-ERBB3 dimers in DFCI81, DFCI161, HCC827, and HCC827GR6 cells. Bar graphs represents pooled data from biological replicate studies of DFCI81 and DFCI161, with each bar depicting mean and standard deviation. Dimerization frequencies displayed were calculated by assessing the average intensity of ERBB3 IP-MET IB or ERBB3 IP-EGFR IB and normalizing through division by the average intensity of ERBB3 IP-ERBB3 IB for each pulldown. Normalization permitted quantification of the association of a proportion of ERBB3 with each receptor, avoiding the confounding effects of ERBB3 expression fluctuation induced by inhibitor treatment.

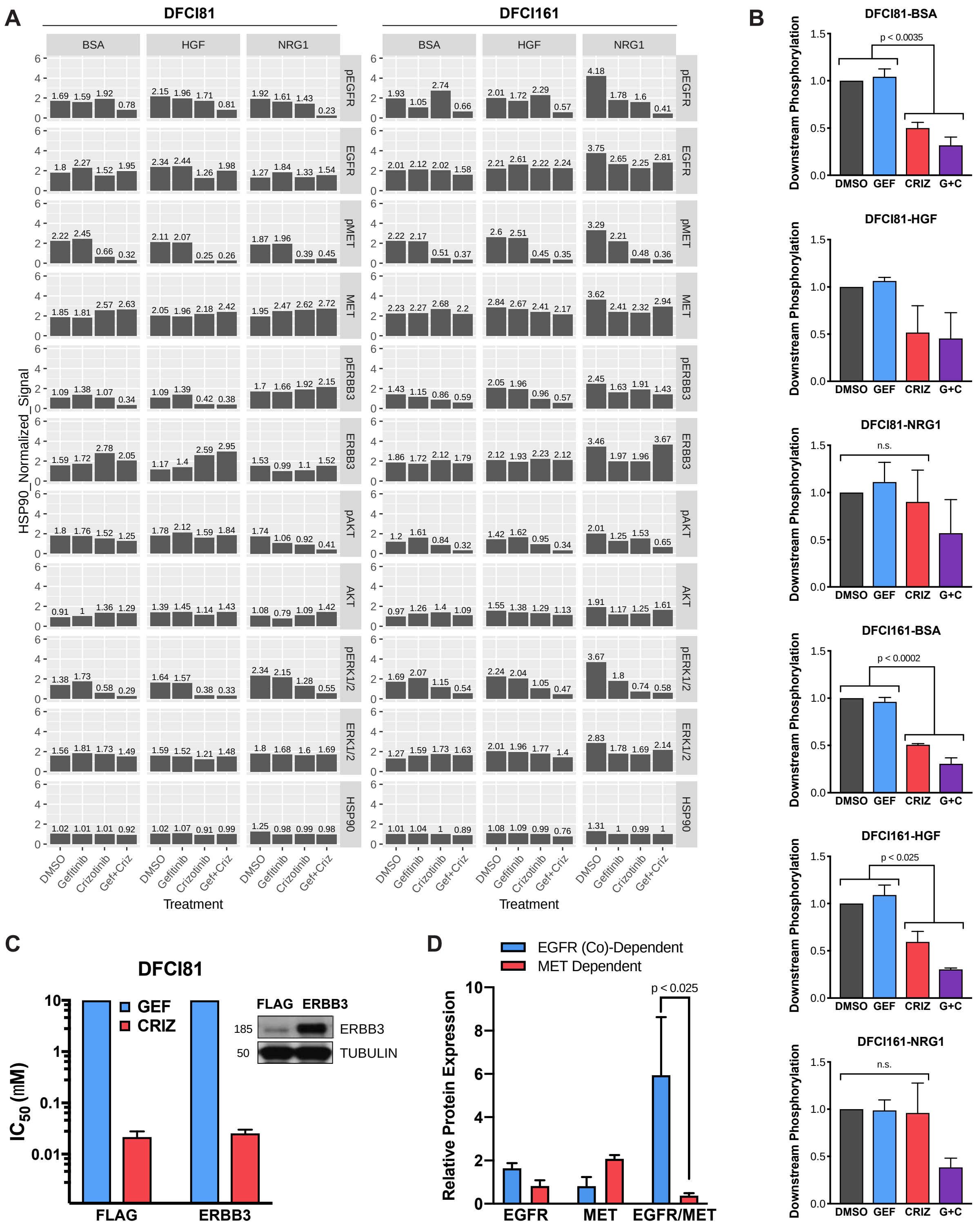

Figure S6: Impact of ERBB3 modulation on drug sensitivity and downstream signaling and EGFR/MET protein quantification in EGFR- and MET-dependent cell line panel.

(A) HSP90 loading control-normalized quantification of Western blot in Fig. 3C. **(B)** Comparison of pooled downstream signaling following EGFR and MET inhibitor treatment in the presence or absence of MET- and ERBB3-activating ligands. Quantified downstream phospho-proteins (pERBB3, pAkt, pERK1/2) were normalized to HSP90, coinciding total protein, and DMSO. Statistical significance was determined by one-way ANOVA followed by Tukey's multiple comparisons post-test. **(C)** Quantification of drug sensitivity in DFCI81 cells transduced to ectopically overexpress of ERBB3 versus empty vector control (FLAG). Cell viability assay was completed following 96-hour incubation in gefitinib or crizotinib. Each bar represents the mean and standard deviation of the IC_{50} derived from three replicate CellTiter-GLO viability experiments. Western blot to corroborate upregulation of ERBB3 protein shows representative data run alongside the viability assay. **(D)** Pooled comparison of EGFR and MET protein expression and EGFR/MET protein expression ratio in EGFR-dependent and -co-dependent cell lines (PC9, H3255, HCC827, HCC827GR6) versus MET-dependent cell lines (DFCI81, DFCI161, EBC-1, H1975). β-actin-normalized EGFR and MET protein values and their ratios were plotted, and statistical significance was determined by one-way ANOVA followed by Tukey's multiple comparisons post-test.

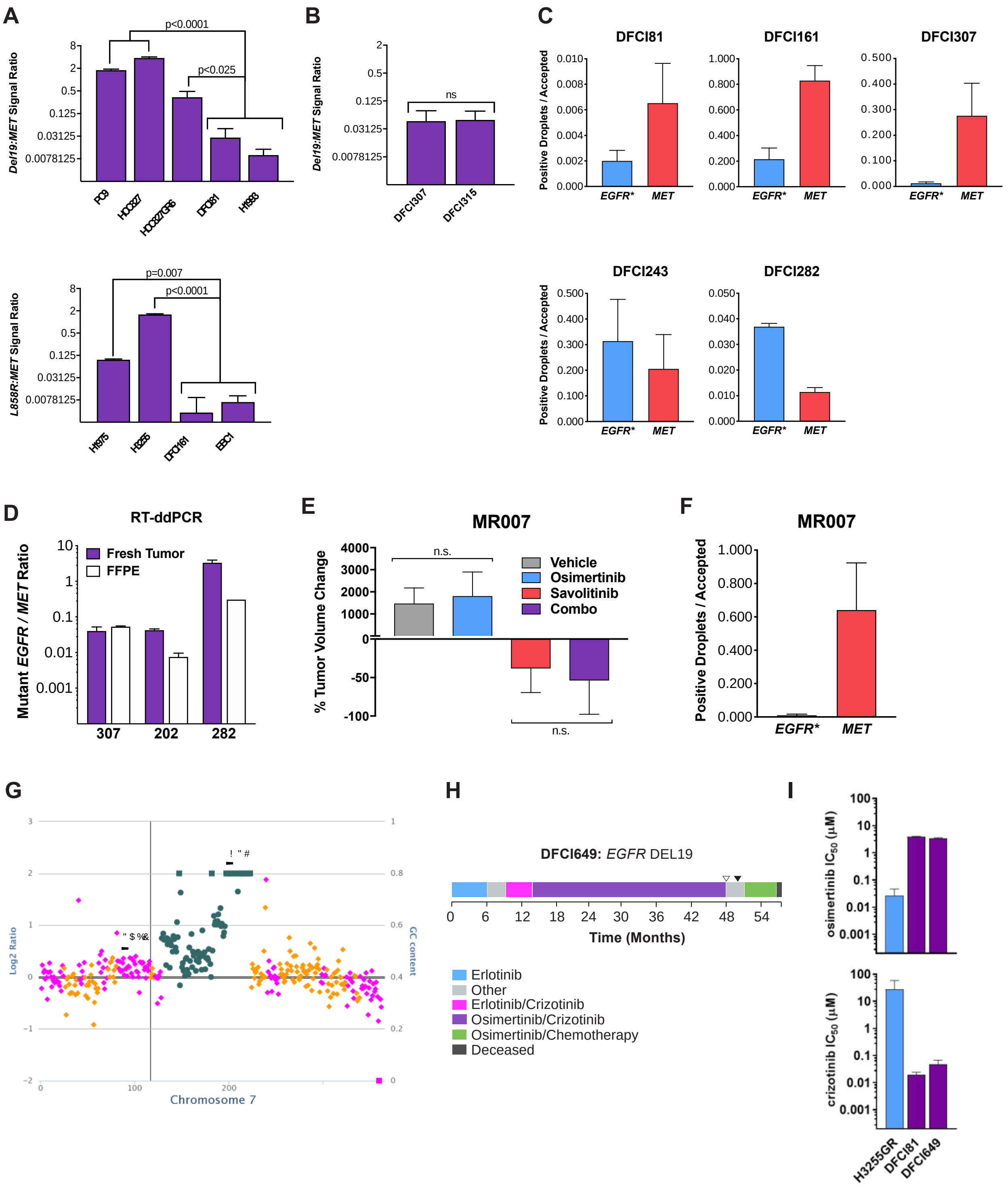

Figure S7: BaseScope and RT-ddPCR analysis, and characterization of additional patient-derived *EGFR*-mutant, MET-dependent NSCLC models.

(A) Ratios calculated from BaseScope *in situ* RNA quantification reveal the mutant EGFR:MET expression ratios in *EGFR*-mutant, MET-dependent DFCI81 and DFCI161 cells compared to EGFR-dependent (PC9, HCC827, H1975, H3255), EGFR/MET-co-dependent (HCC827GR6), and MET-dependent (H1993, EBC-1) controls. Mutant EGFR:MET expression ratios were calculated for each of four quantified fields of view. Statistical significance and p-values were assessed by ANOVA analysis, followed by Holm-Šídák post-test for multiple comparisons. **(B)** *EGFR*-mutant, MET-dependent DFCI307 PDX tumors exhibit comparable and statistically indistinguishable EGFR Del19:MET ratio to *ERBB2*-mutant, *EGFR* wild type, EGFR-independent DFCI315 tumors, as determined by BaseScope analysis. Mutant EGFR:MET expression ratios were calculated for each of four quantified fields of view. Statistical significance was assessed by ANOVA analysis, followed by Holm-Šídák post-test for multiple comparisons. **(C)** Raw values for mutant *EGFR* and *MET* transcript expression by RT-ddPCR in the *EGFR*-mutant, MET-dependent PDX models DFCI81, DFCI161, and DFCI307 compared to the *EGFR*-mutant, EGFR-dependent DFCI243 and DFCI282 PDX models. **(D)** Comparison of mutant *EGFR/MET* transcript stoichiometry ratios measured by RT-ddPCR in fresh tumors versus formalin-fixed, paraffin-embedded specimens for three PDX models. Bars represent mean and standard deviation from technical duplicate assays. **(E)** Single agent MET TKI savolitinib treatment is statistically indistinguishable from combination EGFR and MET kinase inhibitor response in *EGFR*-mutant, MET-driven MR007 PDX model. Results from the waterfall plots shown in Fig. 5A were pooled, and a lack of statistical significance between treatment arms was determined by one-way ANOVA followed by Tukey's post-test for multiple comparisons. **(F)** RT-ddPCR quantification of mutant *EGFR* and total MET transcripts in the *EGFR*-mutant, MET-dependent MR007 patient-derived xenograft model. **(G)** Chromosome 7 oncoplots from

patient-derived sample showing *MET* copy number in DFCI649. **(H)** Treatment history of the patient whose tumor specimen gave rise to the DFCI649 organoid model. White arrow indicates time of specimen collection for OncoPanel sequencing; black arrow indicates time of specimen collection for organoid model establishment. **(I)** Osimertinib and crizotinib sensitivity of DFCI649 organoid model compared to EGFR-dependent H3255GR and MET-dependent DFCI81 organoid control models.

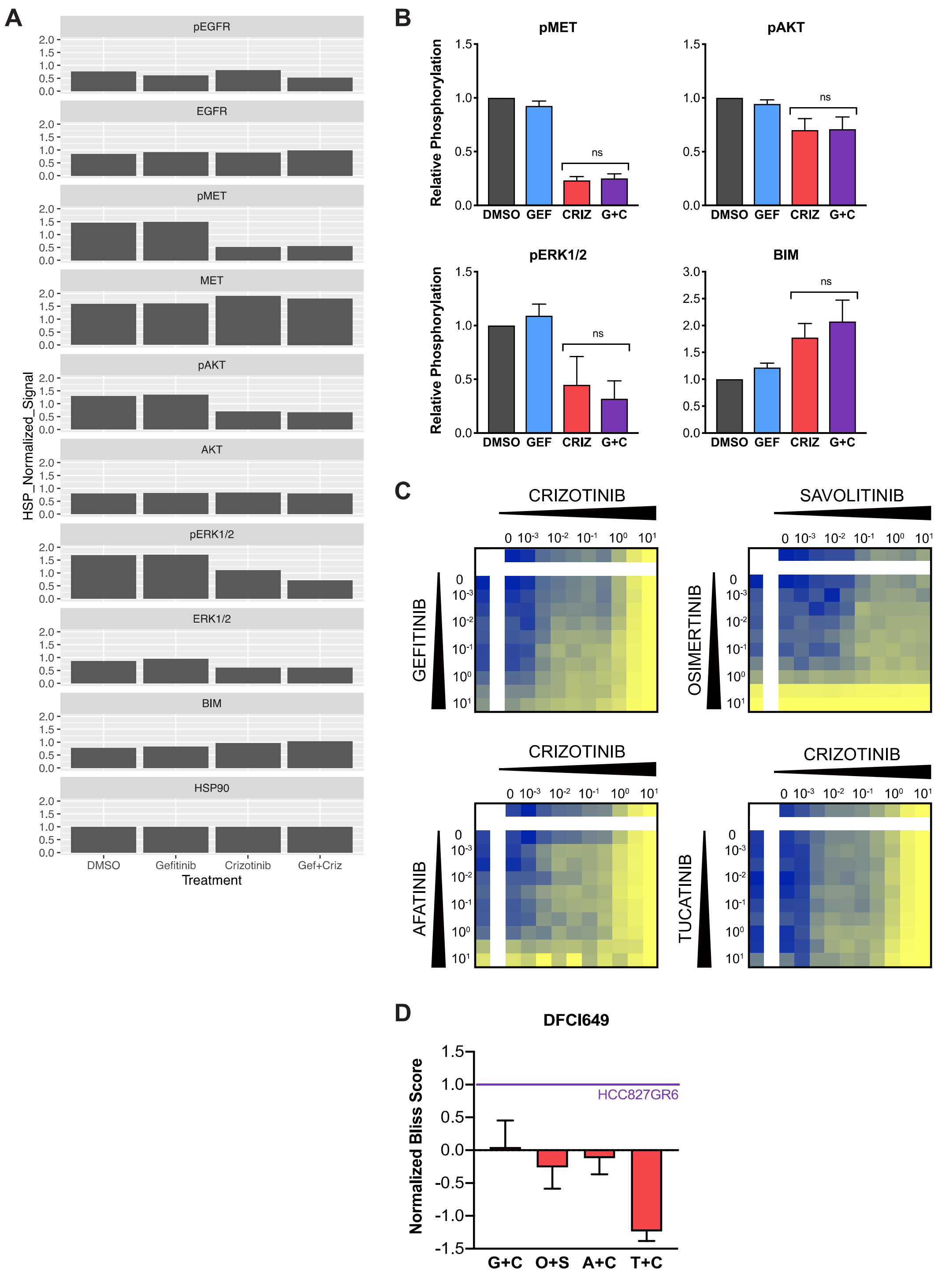

Figure S8: Characterization of DFCI649 patient-derived model of single-agent MET-dependency in *EGFR*-mutant NSCLC.

(A) HSP90 loading control-normalized quantification of Western blot from Fig. 5D. **(B)** Pooled analysis reveals that the magnitude of regulation of phosphoproteins and BIM in DFCI649, DFCI81, and DFCI161 cells by single-agent crizotinib versus combination of gefitinib and crizotinib does not differ significantly. Quantified proteins were normalized to HSP90, with phosphoproteins additionally normalized to coinciding total protein. DMSO-normalized values are plotted for each protein. Statistical significance was assessed by one-way ANOVA followed by Tukey's multiple comparisons post-test. **(C)** Synergy matrices combining targeted EGFR/ERBB inhibitors (vertical gradient) and MET inhibitors (horizontal gradient reveal sensitivities of DFCI649 organoid cells. **(D)** Bliss synergy scores for the combination of EGFR/ERBB and MET were calculated as previously described *(23)* and normalized for comparison to the EGFR/MET-co-dependent HCC827GR6 cell line.

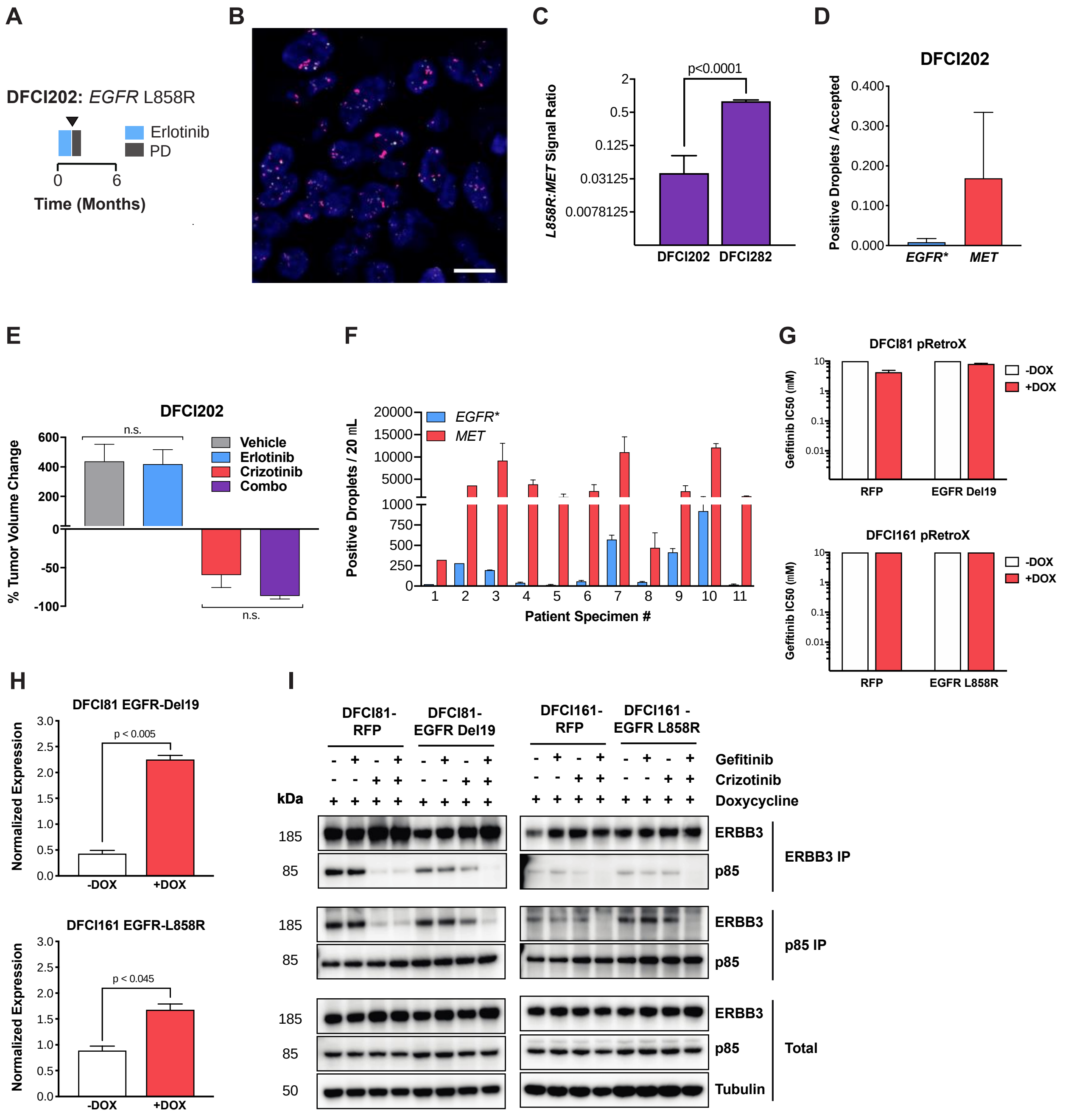

Figure S9: Extended characterization of additional patient-derived *EGFR*-mutant NSCLC models predicted to be MET-dependent, and an analysis of the effects of inducible mutant *EGFR* overexpression in DFCI81 and DFCI161 cells.

(A) Treatment history of a patient whose tumor harbored an activating *EGFR* L858R mutation and concurrent *MET* amplification. Patient-derived xenograft model established from the tumor

of this patient was designated DFCI202. **(B)** FISH assay for genomic MET (red) and centromere 7 (yellow) of a DFCI202 patient-derived xenograft tumor reveals *MET* amplification. The inset MET value for each FISH panel represents the mean MET copy number per cell. Scale bar indicates a length of 20 µm. FISH image was captured using a Nikon Eclipse 80i Fluorescence Microscope. **(C)** *EGFR*-mutant, MET-amplified DFCI202 PDX tumors exhibit significantly lower EGFR L858R:MET ratios compared to EGFR-dependent DFCI282 cells, as determined by BaseScope analysis. Mutant EGFR:MET expression ratios were calculated for each of four quantified fields of view, and data shown are from one of two representative tumors from different animals that were analyzed. Statistical significance and p-values were assessed by ANOVA analysis, followed by Holm-Šídák post-test for multiple comparisons. **(D)** RT-ddPCR analysis to quantify transcript expression of mutant *EGFR* and MET in the *EGFR*-mutant, MET-amplified DFCI202 PDX model. **(E)** Single-agent MET TKI crizotinib treatment was statistically indistinguishable from combination EGFR and MET kinase inhibitor response in the *EGFR*-mutant, MET-driven DFCI202 PDX model. Results from the waterfall plots shown in Fig. 5F were pooled, and a lack of statistically significant difference between treatment arms was determined by one-way ANOVA followed by Tukey's post-test for multiple comparisons. **(F)** Range of mutant *EGFR* and *MET* transcript expression detected by RT-ddPCR among patient tumors harboring concurrent *EGFR* mutation and *MET* amplification. Where there was sufficient material, assay was repeated three times and the bars represent the mean and standard error of the mean between replicate studies. **(G)** Effect of doxycycline-inducible overexpression of EGFR Del19 in DFCI81 cells and of EGFR L858R in DFCI161 on gefitinib resistance. **(H)** Doxycycline-mediated upregulation of mutant EGFR protein in transduced DFCI81 and DFCI161 cells. Plot represents quantification of Western blot in Fig. 6B. Statistical significance

was evaluated by paired t-test. **(I)** Co-immunoprecipitation studies to assess the effects of ectopically overexpressing mutant *EGFR* on the oncogene dependence of DFCI81 and DFCI161 cell lines. Oncogene dependence of the transduced cell lines can be inferred by the inhibitors required to disrupt the interaction between ERBB3 and the p85 subunit of PI3K. DFCI81 and DFCI161 pRetroX-RFP and pRetroX-EGFR cells were seeded in 2.5 µg/mL doxycycline and treated the following day with 0.5 µM of the specified kinase inhibitor. Cells were lysed following 8 hours of treatment in inhibitor and immunoprecipitation was completed on fresh, unfrozen lysates. Blot displayed is representative of three replicate studies, and quantified in Fig. 6C.

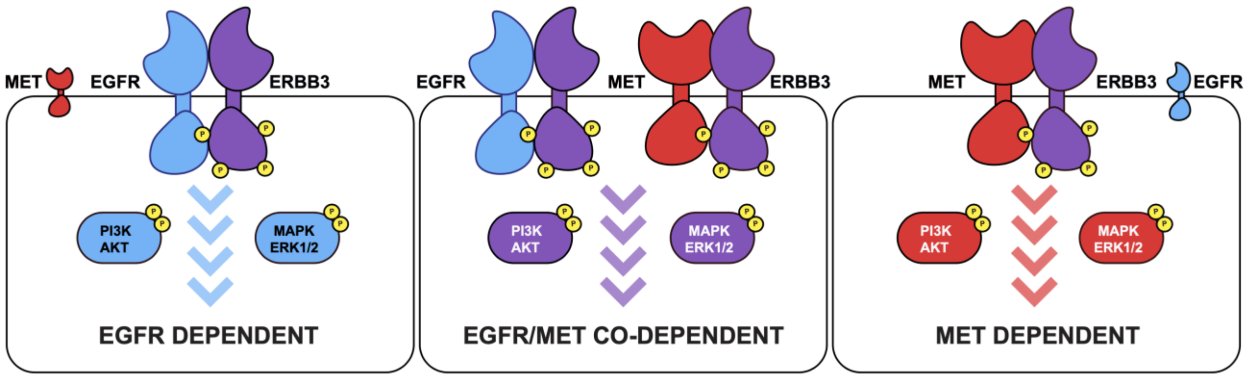

Figure S10: Oncogene dependences in *EGFR*-mutant, *MET*-amplified NSCLC.

In contrast to *EGFR*-mutant lung cancers that exhibit EGFR dependence and *EGFR*-mutant, *MET*-amplified lung cancers that develop EGFR/MET co-dependence, a subset of *EGFR*-mutant NSCLC shows MET dependence accompanied by downregulated EGFR transcript and protein expression and preferentially MET-mediated activation of downstream kinase cascades.

Table S1. Sequences of plasmids and oligonucleotides

Oligo	Function	Sequence or Company/Catalog Number
Primers	EGFR CRISPR-Seq Amplification Primers	F: TTCGGCACGGTGTATAAGGG<br>R: GACATAGTCCAGGAGGCAGC
	EGFR Sanger Sequencing Primers	AAAACCGGACTGAAGGAGCT<br>CGTGAAGAAGTGTCCCCGTA<br>GGAGATAAGTGATGGAGATGTGA<br>CATCCAAACTGCACCTACGG<br>GCTCATCACGCAGCTCATG
	Retro-X Tet-One Cloning Primers	F: GAGGTGGTCTGGATCATGCGACCCTCCGGGACG<br>R: CCCTCGTAAAGAATTTGCTCCAATAAATTCACTGCTTTG
qRT-PCR Primers	EGFR (FAM)	Life Technologies, 4251370: Hs01076078
	MET (FAM)	Life Technologies, 4251370: Hs01565584
	ACTB (VIC)	Life Technologies, 4448490: Hs99999903_m1
	GUSB (VIC)	Life Technologies, 4326320
RT-ddPCR Primers	EGFRex19_f_exp	GGA CTC TGG ATC CCA GAA GG
	EGFRin20.ex19_r_exp	CATCACGTAGGCTTCATCGAG
	EGFRin20.ex21_f_exp	GTGTGCAGATCGCAAAGGGCATG
	EGFRex21_r_exp	CCTCCTTCTGCATGGTATTCTTTCT
	METin18.ex19_f_exp	GCTGCAAGAAACTGTATGCTGG
	MET_r_exp	TGCACCTGTTTTGTTGTGTACACTA
RT-ddPCR Probes	ex19 wt (HEX)	AGCGACGGGAATTTTAAC
	ELREA (FAM)	CT ATC AAA ACA TCT CCG
	L858Rwt (HEX)	AGTTTGGCCAGCCCAA
	L858R (FAM)	AGTTTGGCCCGCCCAA
	METwt (FAM)	CTTGCCAGAGACATGTAT
	ex.19 del pt. 3 (FAM)	CTATCAAGGTTCCGAAAG
	ex. 19 del pt.4 (FAM)	CTATCAAGACATCTCCG

Table S2. Clinical characteristics of *MET* amplified EGFR inhibitor-treated patients

Patient	EGFR Mutation	Prior EGFR TKI therapy	MET amplification detection
1	L858R	Erlotinib	Tissue NGS
2	L858R	[1]None	Tissue NGS
3	Exon 19 Del	Gefitinib, osimertinib	Tissue NGS & FISH
4	L858R	Erlotinib	Tissue NGS
5	Exon 19 Del	Gefitinib, osimertinib	Tissue NGS
6	Exon 19 Del/T790M	Erlotinib, osimertinib	Tissue NGS
7	Exon 19 Del	Erlotinib	Tissue NGS
8	L858R/T790M	Erlotinib	Tissue NGS
9	L858R	Erlotinib	Tissue NGS & FISH
10	Exon 19 Del	Erlotinib, ASP82733, osimertinib	Tissue NGS
11	L858R	Osimertinib	Tissue FISH

[1]Patient with de novo *MET* amplification. Del; deletion, NGS; next generation sequencing, FISH; fluorescence in situ hybridization

Table S3. Antibodies, compounds, and reagents

Antibody / Compound / Reagent		Company	Catalog #
Antibody	β-actin	Sigma Aldrich	A3854
	Akt	Cell Signaling Technology	9272
	pS473 Akt		4060
	BIM		2933
	EGFR		4267, 2232
	EGFR Del19		2085
	EGFR L858R		3197
	pY1068 EGFR		3777
	ERK1/2		9102
	pT202/Y204 ERK1/2		4370
	HER3		4754, 12708
	pY1197 HER3		4561
	pY1289 HER3		2842
	MET		8198
	pY1234/5 MET		3126
	Anti-Rabbit IgG - HRP		7074
	HSP90	Santa Cruz Biotechnology	SC-7947
	P85	Millipore Sigma	ABS233
	α-Tubulin	Sigma Aldrich	T9026
	Anti-Mouse IgG - HRP	VWR	95017-332
Tissue Culture Reagents and Supplies	Afatinib	Fisher Scientific	506998
	Crizotinib	Selleck	S1068
	Gefitinib		S1025
	Merestinib		S7014
	Osimertinib		S7297
	Savolitinib	Active Biochem	HMPL-504
	HGF	Fisher Scientific	PHG0254
	NRG1	Abcam	ab50227
	BSA	Bioworld	220700183
	DMSO	Sigma Aldrich	D8418
	Fetal Bovine Serum		F2442
	Tetracycline-Free FBS	Takara Bio	631101
	Doxycycline Hyclate	Sigma Aldrich	D9891
	ACL-4	Fisher Scientific	0890137DJ
	RETM	Cellaria Biosciences	CM0001
	B-27 Serum-Free Supplement	Thermo Fisher Scientific	17504001
	Matrigel	Corning	356231
	DMEM	Life Technologies	11995073
	RPMI-1640		11875119
	Opti-MEM Medium		31985088
	Penicillin-Streptomycin		15140163
	Phosphate-Buffered Saline		10010049
	DPBS		14190250
	Trypsin		25300120
	Ultrapure Water		10977023
	TrypLE Express Enzyme	Thermo Fisher Scientific	12604013
	RBC Lysis Buffer	Boston BioProducts Inc.	IBB-197
	CD45 Depletion Microbeads	Miltenyl Biotex	130-045-801
	96-Well Plates - Clear	Westnet Inc.	353072

	96-Well Plates - White	Fisher Scientific	07200628
	384-Well Plates - White	Corning	3570
	384-Well ULA Plates		4588
	MTS Assay Reagent	Promega	G111
	CellTiter-Glo Reagent		G7573
	CellTiter-Glo 3D Reagent		G9683
	Phenozine Methosulfate	Sigma Aldrich	P9625
	CellEvent Caspase-3/7 Green	Fisher Scientific	R37111
In Vivo Reagents	Matrigel	Corning	354234
	Crizotinib hydrochloride	MedChem Express	HY-50878A
		Aurum Pharmatech	MR70812
	Erlotinib hydrochloride	Selleck	S1023
	HPMC	Sigma	09963
	Captisol	CyDex Pharmaceuticals	RC0C7100
Molecular Biology Reagents	TRIzol	Life Technologies	15596018
	TaqMan Gene Expression Master Mix		4369016
	RNA Isolation Kit	Qiagen	74104
	Reverse Transcription Kit		205313
	Plasmid miniprep Kit		27104
	PCR Purification Kit		28104
	One-Step RT-ddPCR	Bio-Rad	1864021
	Mycoplasma Plus PCR Primers	Agilent	302008
	Phusion HF Polymerase	New England Biolabs	M0532
	Optical 96-Well Plates	Fisher Scientific	AB0600
	OneShot Stbl3 Competent *E. coli*		C737303
	Syringe Filter 0.45 µm		723-2545
	Puromycin		NC9138068
	Syringe	BD	302995
	Retro-X Tet-One System	Clontech Laboratories, Inc.	634307
	In-Fusion HD Cloning Kit	Takara Bio	638909
	Xfect Transfection		631317
	Retro gag/pol Packaging Vector	Addgene	14887
	pMD2.G Envelope Vector		12259
	Polybrene	Santa Cruz Biotechnology	SC-134220
Western Blotting Reagents	MOPS SDS Running Buffer	Life Technologies	NP0001
	4-12% Bis-Tris Gels		WG1402BOX
	HRP Substrate		34076
	RIPA Buffer with Triton X	Boston BioProducts Inc.	BP-116TX
	NP-40 Buffer		BP-431
	Transfer Buffer		BP-190
	SDS Sample Reducing Buffer		BP-111R
	Phosphatase Inhibitor Tablets	Sigma Aldrich	04906837001
	Protease Inhibitor Tablets		11836170001
	Tween20		P7949
	Tris-Buffered Saline	Westnet Inc.	BM-301X
	A/G-Plus Agarose Beads	Santa Cruz Biotechnology	sc-2003
	BCA Assay	Thermo Fisher Scientific	PI23225
	Microcentrifuge Tubes	Thomas Scientific	C2170
	Protein Standards Ladder	Bio-Rad Laboratories	1610394
	Nonfat Milk Powder		1706406
	Methanol	Fisher Scientific	A412P4
	Immobulin-P Membranes		IPVH00010
	BSA	Bioworld	220700183
